# The Regulatory Network of Natural Competence and Transformation of *Vibrio cholerae*

**DOI:** 10.1371/journal.pgen.1002778

**Published:** 2012-06-21

**Authors:** Mirella Lo Scrudato, Melanie Blokesch

**Affiliations:** Global Health Institute, School of Life Sciences, Ecole Polytechnique Fédérale de Lausanne (EPFL), Lausanne, Switzerland; Universidad de Sevilla, Spain

## Abstract

The human pathogen *Vibrio cholerae* is an aquatic bacterium frequently encountered in rivers, lakes, estuaries, and coastal regions. Within these environmental reservoirs, the bacterium is often found associated with zooplankton and more specifically with their chitinous exoskeleton. Upon growth on such chitinous surfaces, *V. cholerae* initiates a developmental program termed “natural competence for genetic transformation.” Natural competence for transformation is a mode of horizontal gene transfer in bacteria and contributes to the maintenance and evolution of bacterial genomes. In this study, we investigated competence gene expression within this organism at the single cell level. We provide evidence that under homogeneous inducing conditions the majority of the cells express competence genes. A more heterogeneous expression pattern was observable on chitin surfaces. We hypothesize that this was the case due to the heterogeneity around the chitin surface, which might vary extensively with respect to chitin degradation products and autoinducers; these molecules contribute to competence induction based on carbon catabolite repression and quorum-sensing pathways, respectively. Therefore, we investigated the contribution of these two signaling pathways to natural competence in detail using natural transformation assays, transcriptional reporter fusions, quantitative RT–PCR, and immunological detection of protein levels using Western blot analysis. The results illustrate that all tested competence genes are dependent on the transformation regulator TfoX. Furthermore, intracellular cAMP levels play a major role in natural transformation. Finally, we demonstrate that only a minority of genes involved in natural transformation are regulated in a quorum-sensing-dependent manner and that these genes determine the fate of the surrounding DNA. We conclude with a model of the regulatory circuit of chitin-induced natural competence in *V. cholerae.*

## Author Summary

The human pathogen *Vibrio cholerae* is an aquatic bacterium often encountered in rivers, estuaries, and coastal regions. Within this environmental niche, the bacterium often associates with the chitinous exoskeleton of zooplankton. Upon colonization of these chitinous surfaces, *V. cholerae* switches on a developmental program known as natural competence for genetic transformation. Natural competence for transformation is a mode of horizontal gene transfer that allows bacteria to acquire new genes derived from free DNA, which is released by other members within the same habitat. The evolutionary consequences could be that the bacterial recipient becomes better adapted to its environmental niche or, in a worst-case scenario, more pathogenic for man. The results of this study show that, under optimal conditions, the majority of cells within a *V. cholerae* population express competence genes. However, in an aquatic environment, a combination of different ecological factors might lead to heterogeneity in the competence phenotype. Therefore, we investigated the role of extracellular and intracellular signaling molecules with respect to competence induction. This report illustrates that at least three interconnected signaling cascades are required for competence induction, which are based on bacterial metabolism and group behavior.

## Introduction

The bacterium *Vibrio cholerae* is a facultative pathogen and the causative agent of the disease cholera. Cholera is far from being extinct and is, in fact, considered a re-emerging disease [1]. The destructive capacity of cholera is demonstrated by its current outbreak in Haiti. According to a recent health bulletin issued by the Ministère de la Santé Publique et de la Population (MSPP) of Haiti and the PAHO, 515'699 cholera cases have been reported from Haiti up to November 30^th^ 2011 with more than 6'942 deaths. This epidemic highlights the fact that new modeling approaches are required to allow for the prediction of cholera outbreaks in time and space [2]–[5]. However, it also initiated discussions on the origin of the *V. cholerae* strain, which appears more closely related to south Asian strains than to Latin American and U.S. Gulf Coast isolates [6]. This study by Chin *et al.* [6] once more reminded us of the differentiation power that whole genome sequencing provides. Indeed an earlier study by Rita Colwell and collaborators compared 23 *V. cholerae* strains isolated over the past 98 years using whole genome sequencing [7]. These authors concluded that “*V. cholerae* undergoes extensive genetic recombination via lateral gene transfer”. It is therefore of major importance to understand the mechanisms underlying horizontal gene transfer (HGT).

Natural competence for transformation, as one of the three modes of HGT in bacteria, describes the physiological state that allows a bacterium to take up free DNA from the environment. If the internalized DNA is recombined into the chromosome, the bacterium is considered naturally transformed. *V. cholerae* commonly occurs in aquatic ecosystems, its true habitat, where it intimately associates with zooplankton and their chitinous exoskeleton [8]–[10]. In this context, it has been shown that chitin, the polymer used as the building block of planktonic exoskeletons, induces natural competence for transformation of *V. cholerae* [11]. Thus, HGT is tightly linked to the environmental niche of *V. cholerae* and potentially also to the niche of many other *Vibrio* species. In fact, three other species of the genus *Vibrio*, *V. fischeri*, *V. vulnificus* and *V. parahaemolyticus*, are naturally transformable in a chitin-dependent manner [12]–[14].

Transforming DNA can be used to repair damaged genes and, therefore, contributes to genome maintenance or to the acquisition of new alleles/genes, which lead to genetic diversity and evolution. Indeed, experimental laboratory microcosm experiments that simulate aquatic environments have succeeded in recapitulating a *V. cholerae* O1-to-O139 serogroup conversion by means of natural transformation [15]. This result provides a potential explanation for the devastating occurrence of the O139 serogroup variant of *V. cholerae*. Today this strain is almost undetectable in endemic regions even though researchers have feared its occurrence as the onset of a new and, therefore 8^th^, cholera pandemic [16]. However, an important lesson can be learned from the emergence of this new strain: by means of horizontal gene transfer (HGT), *Vibrio* species may exchange genetic material and become more virulent to mankind.

Chitin-induced natural competence and transformation is poorly understood in spite of its importance. Based on a few suggestive experiments on how natural competence could be regulated in *V. cholerae*, the authors of a previous study proposed a model that involved at least three regulatory pathways [11]: 1) induction by chitin, 2) catabolite repression, and 3) quorum-sensing (QS). We and others followed up on this study and provided further evidence for an involvement of these three pathways [17]–[23]. However, all of these studies have only looked at a population-wide level. This could lead to a lack of information on how competence is regulated within a single cell. This is exemplified in one of the best-studied naturally competent bacterial species, *Bacillus subtilis*, for which it is known that “a majority of the bacteria being insusceptible and a minority being highly susceptible to transformation” [24]. David Dubnau and collaborators explained why only 10–20% of cells within a *B. subtilis* population enter the competence state, and demonstrated that such bistability is caused by intrinsic noise in competence gene expression [25], [26]. Here we show, for the first time, that under homogeneous competence–inducing conditions *V. cholerae* displays a homogeneous expression pattern as the vast majority of cell within a population scored positive for expression of competence genes.

Taking this important finding into consideration, we then moved on to establish an inducible competence system for *V. cholerae*, which is based on low levels of TfoX production and not on *tfoX* overexpression as previously done. To date all studies on natural competence and transformation in *V. cholerae* have only looked at single genes involved in the competence program, at single pathways, and at varying inter-experimental conditions (e.g., chitin surface transformation phenotypes compared to artificial competence induction with plasmids in rich medium etc.) [17], [20]–[23]. This inducible and chromosomally encoded competence system allowed us to look at different aspects of the regulatory network of natural competence under standardized conditions. Based on these new data, we propose a model of how the regulatory network of natural competence functions in *V. cholerae*.

The goals of our study were to 1) investigate whether natural competence is induced in a whole population under natural or optimized conditions, 2) establish a homogeneous competence-inducing system to investigate the contribution of separate and interconnected regulatory pathways to competence induction, and 3) test whether different competence genes are subject to the same regulatory circuits. We achieved these aims by using transcriptional reporter fusion constructs of representative competence genes. We combined these fluorescent reporter fusions with the following detection methods: epifluorescence microscopy and flow cytometry, which allowed us to visualize the expression of fluorescent reporter genes at the level of single cells and to quantify gene expression accordingly; and fluorescent plate reading, which we used to investigate a plethora of regulatory mutants and regulated genes based on population average fluorescent value measurements.

## Results/Discussion

### Visualization of competence gene expression upon chitin surface colonization

To better understand whether natural competence of *V. cholerae* is a developmental program followed by (almost) all members of a population or rather a state, which only a subpopulation acquires, we investigated gene expression at the single cell level. Therefore, we transcriptionally fused the promoter regions of competence genes to those genes encoding fluorescent proteins (FPs). Our choice of FPs was GFP-mut3* [27] and DsRed.T3[DNT] [28], [29] as both of them have been optimized for fluorescence intensity and can be visualized within the same cell (i.e., the excitation/emission spectra are adequately separated). We initially focused on two promoter regions: the upstream region of the *pilA-D* operon [30], hereafter referred to as the *pilA* promoter, and the region upstream of *comEA*. Both of these genes, *pilA* and *comEA*, are upregulated on chitin ([11], [31]; Blokesch and Schoolnik, unpublished) and essential for natural transformation to occur [11]. *PilA* encodes a major pilin, which, as a part of a hypothetical type IV-like pilus [30], is most likely involved in the DNA uptake process. The same involvement in DNA uptake holds true for ComEA, which shows homology to ComEA of *Bacillus subtilis* [32] as it also contains a helix-hairpin-helix (HhH) motif (pfam12836). HhH motifs have been previously described as short DNA-binding domains that bind DNA in a non-sequence-specific manner [33]. How exactly the DNA uptake, including the involvement of the type IV-like pilus and ComEA, functions is so far unknown for *V. cholerae* and for other naturally competent bacteria.

**Figure 1:**
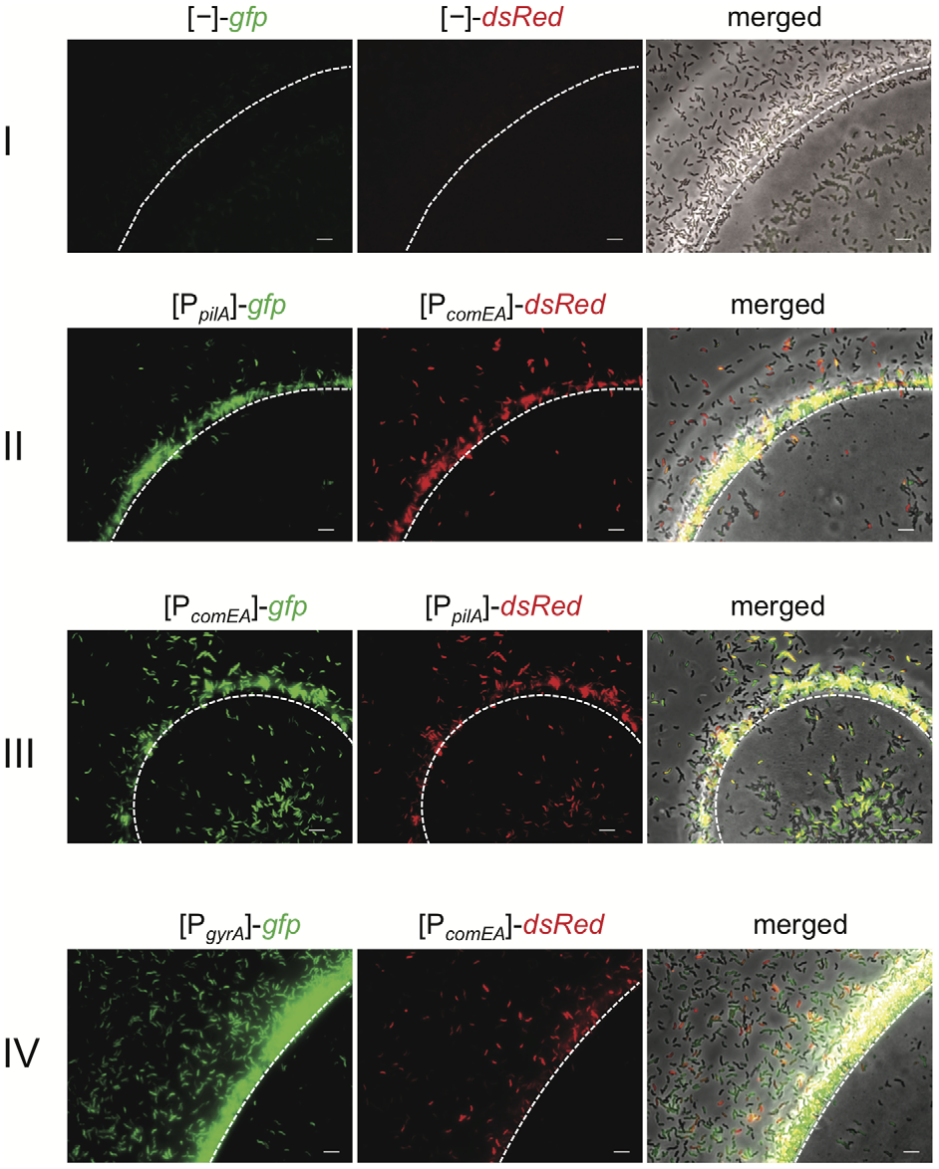
Visualization of competence gene expression on chitin surfaces. *V. cholerae* cells were grown on chitin beads as previously described [23]. The white dashed line notes the edge of the chitin surface. The bacteria carried diverse transcriptional FP reporter fusions. I: vector control containing promoter-less *gfp* and *dsRed*; II: plasmid containing *gfp* driven by the *pilA* promoter and *dsRed* downstream of the *comEA* promoter region; III: swapped reporter genes in comparison to II; IV: plasmid containing *gfp* driven by the *gyrA* promoter and *dsRed* driven by the *comEA* promoter. Bacteria were grown statically for 48 h before pictures were taken. The order of the images here and in the following figures is (from left to right): green channel (GFP), red channel (dsRed), and merged image composed of a phase contrast image overlaid by both fluorescence channel images. Scale bar = 5 µm.

With these reporter fusions in hand, we moved on to visualize competence gene expression. We first tested the expression in *V. cholerae* strains after allowing them to colonize chitin beads. Chitin beads mimic the natural environment of *V. cholerae* in which the bacteria are often found associated with the chitinous exoskeletons of zooplankton [10]. In contrast to other competence-inducing chitin surfaces, such as crab shell fragments or chitin flakes [11], [19], chitin beads are amenable to light microscopy. As shown in Figure 1, no significant green or red fluorescence was detectable by epifluorescence microscopy for *V. cholerae* cells grown on chitin beads if the bacteria carried the promoter-less FP reporter plasmid (vector control; panel I). In contrast, bright fluorescence signals were visible when the FP genes were driven by either of the two promoters belonging to *pilA* or *comEA* (panel II and reciprocal with respect to the FP-fusions in panel III). From the obtained images, it was apparent that not all of the bacteria within the population were fluorescent and, therefore, expressing the competence genes at detectable levels (Figure 1). Based on the finding that not all bacteria appeared as fluorescent we constructed another reporter as positive control, which consisted of the promoter preceeding the housekeeping gene *gyrA* (encoding gyrase) transcriptionally fused to *gfp*. This fusion was cloned onto the same plasmid as the P*_comEA_*-*dsRed* fusion (see Material and Methods). We tested this reporter strain in our chitin bead colonization assay (Figure 1, panel IV). In contrast to *dsRed* being driven by the *comEA* promoter the *gyrA* promoter led to detectable *gfp* expression in a significantly larger fraction of cells. The same expression pattern as for *gyrA* was observable for three additional transcriptional reporter fusions containing the promoter region of *recA*, *clpX*, and *ftsH*, respectively (Figure S1). We included these reporter strains in our study as the expression of the housekeeping gene *gyrA* is most likely controlled by DNA supercoiling as it was demonstrated for *E. coli* [34] and/or might be cell cycle-dependent as shown for *Caulobacter crescentus* [35]. The rational for choosing *recA*, *clpX*, and *ftsH* as additional positive controls was based on the fact that other researchers have already used these genes to normalize quantitative RT-PCR expression data. Furthermore, expression of none of these genes was significantly changed in microarray expression studies using different *V. cholerae* mutant strains ([11], [36] and Blokesch and Schoolnik, unpublished) or using different growth conditions (e.g. comparing rabbit ileal loop grown cells versus exponential *in vitro* cultures of *V. cholerae* [37]).

We considered three different reasons for the finding that competence genes are only expressed at detectable levels in a fraction of the population: 1) competence gene expression in *V. cholerae* is a bistable phenomenon, which is similar to *B. subtilis* [25]; 2) the environment around the chitinous surface is heterogeneous and, thus, does not lead to competence induction in all cells; and 3) the fluorescence signal in cells that appear as non-induced for *pilA* and *comEA* expression is too weak to be detected with our epifluorescence microscopy settings.

To follow up on these three possibilities, we aimed at differentiating between the existence of an intrinsic bistable switch versus the idea of a heterogeneous expression pattern due to heterogeneous conditions and to concomitantly judge whether the seemingly uninduced cells observed on the chitinous surfaces (Figure 1) were the result of experimental limitations in our system.

### Population-wide expression of competence genes under homogeneous conditions

**Figure 2:**
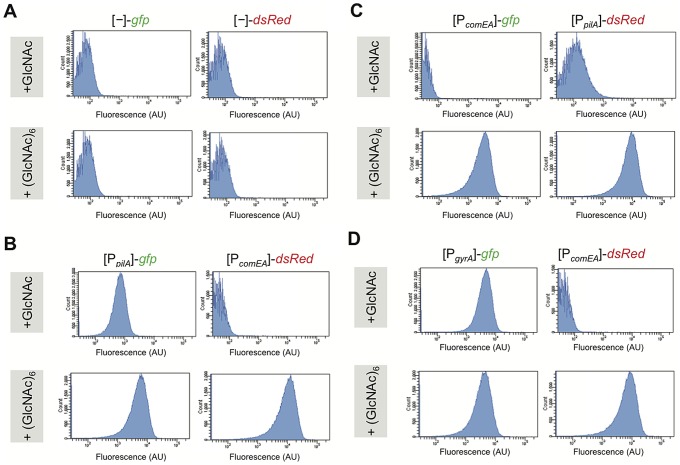
Competence genes are expressed by the majority of cells under homogeneous competence-inducing conditions. *V. cholerae* strains were grown aerobically in defined artificial seawater medium with the addition of either N-acetylglucosamine (GlcNAc) or hexa-N-acetylchitohexaose (GlcNAc)_6_ as sole carbon source. The latter is known as a potent inducer of natural competence/transformation [11]. Competence gene expression was quantified for fluorescence intensities using flow cytometry. Reporter fusions are indicated above each panel. Panel A: promoter-less *gfp* and *dsRed* reporter; panel B: [P*_pilA_*]-*gfp*/[P*_comEA_*]-*dsRed*; and panel C: [P*_comEA_*]-*gfp* and [P*_pilA_*]-*dsRed.* Panel D: [P*_gyrA_*]-*gfp*/[P*_comEA_*]-*dsRed*. The flow cytometry graphs indicate the number of cell counts on the y-axis and the fluorescence signal intensity (as arbitrary units, AU) on the x-axis.

First, we wanted to test if we would observe a bistable competence gene expression pattern under homogeneous growth conditions. Therefore, we changed the chitin substrate from chitin beads (an insoluble GlcNAc polymer) to soluble hexa-N-acetylchitohexaose (from here on referred to as GlcNAc_6_). This chitin oligomer has been used before to induce natural competence in *V. cholerae* [11], [23]. As a control, we grew the same *V. cholerae* reporter strains under identical minimal medium conditions (defined artificial seawater medium, DASW; [11]) but changed the main carbon source from GlcNAc_6_ to the non-competence inducing chitin monomer N-acetylglucosamine (GlcNAc). All strains were grown to the same optical density before being either visualized by epifluorescence microscopy (Figure S2) or quantified with respect to their fluorescence intensity using flow cytometry (Figure 2). As shown in Figure S2A and S2B, we did not detect significant fluorescent signals for the promoterless reporter control using microscopy, which is in accordance with the low levels of fluorescent signals measured by flow cytometry (Figure 2A). Thus, the fluorescence intensity of these bacteria (panel A, flow cytometry graphs) was considered background. For the strains grown with GlcNAc, FP expression driven by the *comEA* promoter was not detectable using microscopy (Figure S2C, S2E, S2G including the respective image analysis) and quantified as extremely low fluorescent signals using flow cytometry (Figure 2B–2D upper row). However, weak *pilA* promoter-driven *gfp* expression in the presence of GlcNAc was observed after an extended exposure time (Figure S2C). We confirmed this basal *pilA* promoter-driven *gfp* expression in the presence of GlcNAc by flow cytometry (mean FU = 7.2×10^2^; Figure 2B, upper row). Swapping the FP reporter gene behind the *pilA* promoter from *gfp* to *dsRed* resulted in undetectable red fluorescence using our epifluorescence microscopy settings (Figure S2E); however, an increased *pilA* promoter-driven expression of *dsRed* compared to the promoterless reporter plasmid control (Figure 2A) was detectable by flow cytometry (Figure 2C, upper row), confirming the low level of *pilA* expression under non-competence inducing conditions.

In cells grown under competence-inducing conditions (e.g. in the presence of GlcNAc_6_), the reporter strains displayed strong *pilA* and *comEA* promoter-driven fluorescent signals (Figure S2D, S2F, S2H). This was the case for the majority of the cells and only a minority appeared as non-fluorescent under these conditions. There was a distribution of fluorescence intensities as depicted in the flow cytometry graphs (Figure 2B–2D, lower row) but the distribution was not bimodal. To further investigate whether the non-fluorescent-appearing cells in the microscopy images (Figure S2) were meaningful with respect to competence expression, we again investigated the behavior of the housekeeping gene reporter strains under these homogenous competence-inducing conditions (for *gyrA* see Figure 2D and Figure S2G and S2H; or for *recA*, *clpX*, and *ftsH* see Figure S3). The same expression pattern as for the competence genes was observed for these strains with a minority of cells not displaying any detectable fluorescence using our epifluorescence microscopy and image display settings. Thus, and also based on the image analysis (Figure S2) and on the flow cytometry measurements (Figure 2), this minority of non-fluorescent-appearing bacteria probably corresponds to cells, which fluoresce at lower levels (mostly with a good correlation between both FPs; Figure S2).

An issue that we had to consider was the fact that our FP reporter constructs were plasmid-encoded. Indeed, plasmid copy numbers can change in *V. cholerae* according to growth rate [38]. However, in our experiments we mainly looked at different strains but under similar growth conditions (and the biological replicates were highly reproducible; Figure S4). Furthermore, a recent study by Silander *et al*. provided evidence that plasmid-based systems are useful to study gene expression in bacteria; they observed that both the mean and the variation of expression correlated well between both settings [39].

### A chromosomally encoded inducible competence system allows quantification of gene expression

**Figure 3:**
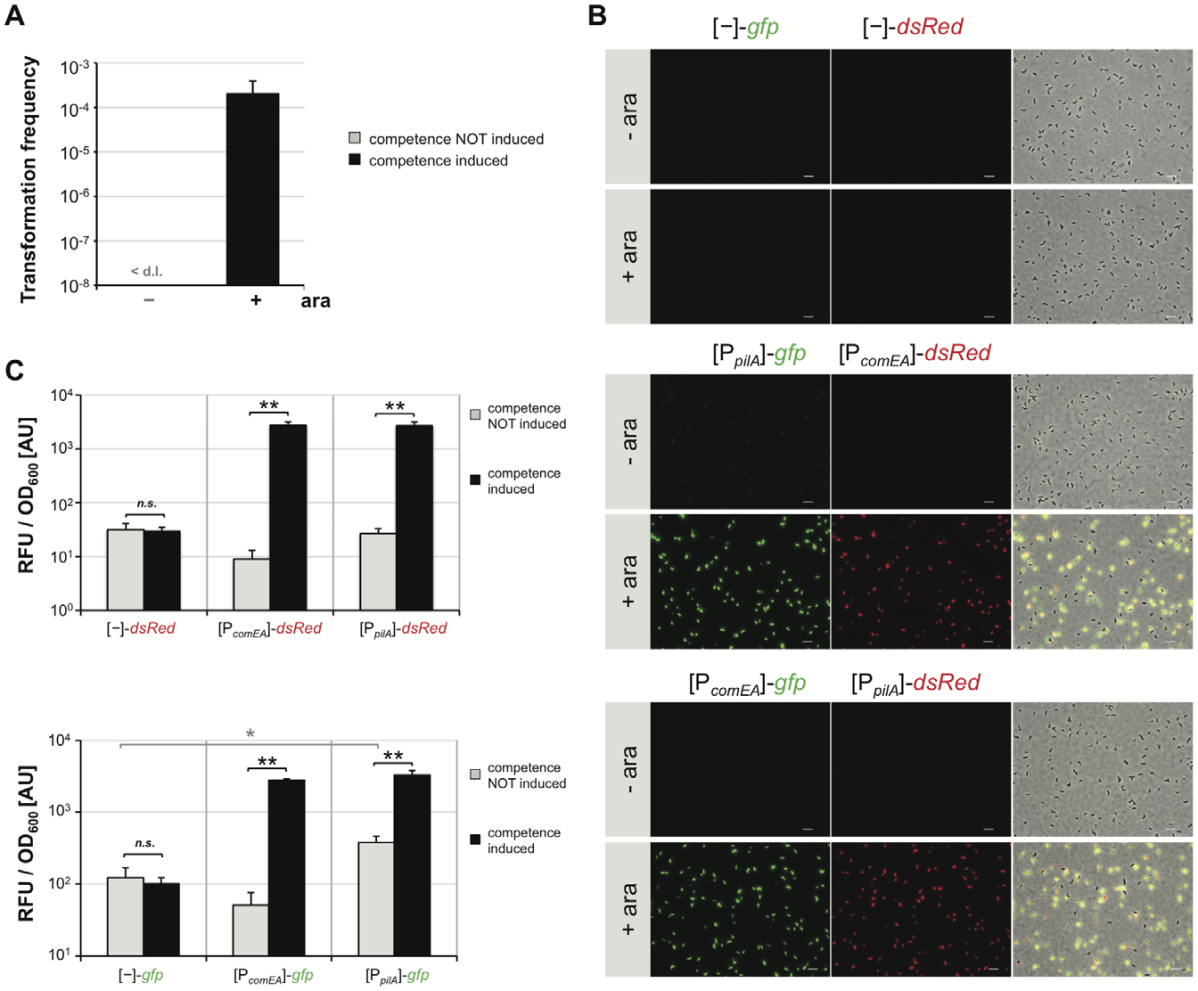
Artificial induction of natural transformation by expression of the competence regulatory gene *tfoX in cis*. *V. cholerae* cells were grown in rich medium in the absence (−) or presence (+) of the artificial inducer arabinose (0.02%). Cells were either tested for natural transformability (panel A) or competence gene promoter activity based on FP reporters (panels B and C). Panel A: Transformation frequencies are given on the y-axis for competence-uninduced (−) and competence-induced (+) bacteria. <d.l. = below detection limit. Panel B and C: *V. cholerae* cells harboring the different transcriptional reporter fusions were grown without or with competence induction. Bacteria were either visualized by epifluorescence microscopy (panel B; image arrangements as in Figure 1; Scale bar = 5 µm) or measured with respect to relative fluorescence units (RFU) and optical density at 600 nm (panel C). Panel C: RFU per OD_600_ values are given on the y-axis. All experiments in Figure 3 were repeated at least three independent times. Error bars reflect standard deviations. Statistics were applied using the Student's *t* test. * *P*<0.05, ** *P*<0.01, *n.s.* = not significant.

Based on the data described above, we concluded that bistability of competence gene expression is unlikely in a population of *V. cholerae* cells under homogeneous competence-inducing conditions. Therefore, it seemed feasible to further investigate the regulatory circuit at a population-wide level given that homogeneous, competence-inducing growth conditions were provided. Unfortunately, GlcNAc_6_, a competence-inducer, has recently been discontinued by the Seikagaku Corporation, and multiple and large-scale experiments using this commercially available compound are extremely costly. Furthermore, shorter GlcNAc oligomers such as GlcNAc_2_ often result in large variations with respect to transformation frequencies (M. Blokesch, unpublished), which is most likely due to GlcNAc monomer impurities within the preparation that exert catabolite repression on natural transformation [23]. Therefore, we thought of establishing a chitin-independent, competence-inducing system. One possibility was to artificially overexpress the major regulator of transformation, TfoX, from a plasmid as previously performed [11], [17], [22], [23]. However, we observed major disadvantages using this method. First, the morphology of some of the cells changed towards a filamentous form after competence induction due to the plasmid-maintaining antibiotic ampicillin (Blokesch, unpublished). Second, TfoX overexpression from a multi-copy plasmid resulted in the induction of heat shock proteins and chaperones [11], which might be indicative of stress conditions. Indeed, the toxicity of TfoX overexpression has been previously described for *Escherichia coli* and *Haemophilus influenzae* [40], [41]. Third, we wanted to avoid working with *V. cholerae* cells containing two different plasmids within the same cell (*tfoX* and FP reporter fusions-carrying). Therefore, we constructed a chromosomally encoded competence induction system, which is based on inducible low-level TfoX production. The system was composed of *tfoX* under the control of an arabinose-inducible promoter (P*_BAD_*) and the gene encoding AraC, which act as a repressor or initiator of gene expression in the absence or presence of L-arabinose, respectively [42]. Both of these elements were cloned into a mini-Tn7 transposon [43], which integrates into the large chromosome of *V. cholerae* (later referred to as Tn*tfoX*). We transferred this transposon by triparental mating into the *V. cholerae* wild type strain A1552 and tested the respective strain for natural transformability in LB medium (Figure 3). By adding a low amount of L-arabinose (0.02%), we obtained transformation frequencies that were two orders of magnitude higher than what has been described for overproduced *tfoX* in *V. cholerae* [11] (Figure 3A). Furthermore, the transformation frequency (2.1×10^−4^) was in the same range as the frequencies we usually obtain using optimized chitin-inducing conditions (3.1×10^−4^; [19]). We were unable to detect any naturally transformed colony-forming units in the inducer-free control culture (shown for the wild type in Figure 3A but likewise tested in all other strains). We also examined the abundance of TfoX at the protein level. To do so we grew the *V. cholerae* strain A1552-Tn*tfoX* in LB medium in the absence or presence of different L-arabinose concentrations followed by western blot analysis of cellular proteins using antibodies against TfoX (Figure S5). In parallel we grew a strain containing inducible *tfoX* on a plasmid similar to the *tfoX*-overexpression system described earlier [11]. As shown in Figure S5 we observed a major difference in TfoX protein levels comparing the previous [11] and current experimental setup as indicated by the two arrows. This reassured us that this system was not heavily overproducing TfoX and was therefore adequate for further analysis to establish the genetic interactions downstream of TfoX.

We first wanted to visualize competence gene expression in this chitin-independent system. We transferred the respective FP reporter fusion constructs into a wild type *V. cholerae* strain carrying the chromosomally encoded *tfoX* construct (A1552-Tn*tfoX*) and visualized FP gene expression by epifluorescence microscopy (Figure 3B). As can be appreciated from the images in Figure 3B (middle and lower part), the *pilA*- and *comEA* promoter-driven expression pattern of the FP reporters under such chitin-independent, competence-inducing conditions mirrored what we observed under the GlcNAc_6_-mediated induction of competence (Figure S2). As described above for the chitin-dependent experiment, only a minority of cells did not display any detectable fluorescence using this microscopy technique, which was also the case for *gyrA* promoter-driven FP reporter expression (data not shown). The fluorescent signal was below the detection limit in cells grown in the absence of inducer (Figure 3B, -ara) or in cells harboring the promoter-less plasmid as a control (Figure 3B, upper two rows).

The fluorescent signal was quantified using a 96-well plate reader (Figure 3C). A statistically significant increase in fluorescence intensity was observed upon induction of competence for all promoter-driven FP reporter fusion constructs (Figure 3C, middle and right columns). No significant difference in fluorescence signals between competence-uninduced and competence-induced conditions was observed for the promoter-less FP reporter control (Figure 3C). In agreement with the chitin data described above, we detected a statistically significant increase in *pilA*-driven *gfp* expression compared to the promoter-less construct even in the absence of inducer. Therefore, we conclude that this basal expression of *pilA* is TfoX-independent.

### Induction of natural competence is dependent on the cAMP level within cells

We then moved on to investigate the regulatory network of natural competence in *V. cholerae* in further detail. The first assay aimed at testing whether carbon catabolite repression (CCR) plays a role in this chitin-independent setup. Carbon catabolite repression occurs if preferred phosphoenolpyruvate∶carbohydrate phosphotransferase system (PTS)-transported sugars are abundant. However, in their absence the PTS systems are unsaturated and indirectly lead to the activation of the enzyme adenylate cyclase, which subsequently synthesizes cAMP within the cell (for review on CCR see [44]). We were mostly interested in *V. cholerae* strains that are impaired in this synthesis or in the degradation of cAMP. The concentration of cAMP within cells is accomplished by interplay between adenylate cyclase (CyaA) and cAMP-degrading phosphodiesterases (CpdA). A recent study by Kim *et al*. demonstrated the importance of CpdA in balancing the intracellular cAMP level in *Vibrio vulnificus* [45]. As the *cpdA* gene of *V. cholerae* is located at the same chromosomal locus (as analyzed using *SynTView*, a synteny viewer developed by the Genomic and Genetic Department of Institute Pasteur, Paris) and *cpdA*/CpdA display 64%/68% identity (76%/82% similarity) at the DNA and protein levels, respectively, the functionality of the protein is most likely identical in both organisms. To disrupt the equilibrium between cAMP production and degradation, the *cpdA* gene in *V. cholerae* was deleted (Table 1). Although the production of cAMP does not change in this mutant, cAMP degradation should be impaired, resulting in the accumulation of cAMP within the cell. We tested this strain in a chitin surface colonization assay [23] and observed a hyper-colonization phenotype consistent with increased intracellular cAMP levels (data not shown). More importantly, we transferred the Tn*tfoX* transposon into this *V. cholerae* strain as well as a strain lacking adenylate cyclase (Δ*cyaA*) and tested both strains with respect to natural transformability (Table 2) and *pilA*/*comEA* promoter-driven FP gene expression (Figure S6). The results confirmed part of what we had previously demonstrated on chitin surfaces [23], namely that adenylate cyclase is essential for natural transformation even under rich culture medium conditions. However, in this study we extended this knowledge by showing that a statistically significant increase in natural transformability occurred in the newly constructed *cpdA* mutant compared to the wild type parental strain (Table 2). With respect to competence gene induction, *pilA* and *comEA* promoter-driven FP gene expression was abolished in the absence of cAMP (Figure S6). This was in contrast to the fluorescent signal measured for the *cpdA* mutant, that is, both the *pilA* and the *comEA* promoter efficiently drove FP gene expression in this genetic background upon competence induction (Figure S6). These data highlight the necessity of cAMP for competence gene expression even when competence induction is uncoupled from chitin surface colonization and chitin degradation (e.g., from metabolism of carbon sources).

**Table 1:** Bacterial strains and plasmids.

Strains or plasmids	Genotype*	References
**Strains** **(** ***V. cholerae*** **)**		
A1552	Wild type, O1 El Tor Inaba, Rif^R^	[68]
A1552-LacZ-Kan	A1552 strain with *aph* cassette in *lacZ* gene; Rif^R^, Kan^R^	[19], [69]
ΔcyaA	A1552ΔVC0122, Rif^R^	[23]
ΔcpdA	A1552ΔVC2433, Rif^R^	this study
ΔcqsA	A1552ΔVC0523, Rif^R^	[22]
ΔluxS	A1552ΔVC0557, Rif^R^	[70]
ΔcqsAΔluxS	A1552ΔVC0523ΔVC0557, Rif^R^	[22]
ΔhapR	A1552ΔVC0583, Rif^R^	[11]
A1552-Tn*tfoX*	A1552 containing mini-Tn7-*araC*-P*_BAD_*-*tfoX*; Rif^R^, Gent^R^	this study
ΔcyaA-Tn*tfoX*	A1552ΔcyaA containing mini-Tn7-*araC*-P*_BAD_*-*tfoX*; Rif^R^, Gent^R^	this study
ΔcpdA-Tn*tfoX*	A1552ΔcpdA containing mini-Tn7-*araC*-P*_BAD_*-*tfoX*; Rif^R^, Gent^R^	this study
ΔcsqA-Tn*tfoX*	A1552ΔcsqA containing mini-Tn7-*araC*-P*_BAD_*-*tfoX*; Rif^R^, Gent^R^	this study
ΔluxS-Tn*tfoX*	A1552ΔluxS containing mini-Tn7-*araC*-P*_BAD_*-*tfoX*; Rif^R^, Gent^R^	this study
ΔcqsAΔluxS-Tn*tfoX*	A1552ΔcqsAΔluxS containing mini-Tn7-*araC*-P*_BAD_*-*tfoX*; Rif^R^, Gent^R^	this study
ΔhapR-Tn*tfoX*	A1552ΔhapR containing mini-Tn7-*araC*-P*_BAD_*-*tfoX*; Rif^R^, Gent^R^	this study
**Plasmids**		
pBR322	Amp^R^, Tc^R^	[61]
pGP704-Sac28	Suicide vector, ori R6K *sacB*, Amp^R^	[31]
pGP704-28-SacB-ΔcpdA	pGP704-Sac28 with a gene fragment resulting in a	
351-bp deletion of VC2433 (*cpdA*)	this study	
pVSV209	Kan^R^, *rfp* (DsRed.T3[DNT]), transcriptional terminators-(*Avr*II, *Sal*I, *Stu*I)-promoterless Cm^R^ and *gfp;* oriV_R6K_;	[29]
pBKdsGFP	Derivative of pBR322; promoterless *gfp* preceded by MCS; Kan^R^, *rfp* (DsRed.T3[DNT]), transcriptional terminators-(*Avr*II, *Sal*I, *Stu*I) derived from pVSV209	this study
pBR-Tet_MCSI	pBR322 derivative deleted for Tet promoter and part of *tet* ^R^ gene; Amp^R^	this study
pBR-Tet_MCSI-GFP	Promoterless *gfp* from pBKdsGFP cloned into *Aat*II/*EcoR*I site of pBR-Tet_MCSI; Amp^R^	this study
pBR-Tet_MCSI-GFP_dsRed	Promoterless *rfp* (DsRed.T3[DNT]) from pBKdsGFP cloned into *Eco*RV/*Bam*HI site of pBR-Tet-MCSI-GFP; Amp^R^	this study
pBR-GFP_dsRed_Kan	*aph* gene (from pBKdsGFP) cloned into *BamH*I site of pBR-Tet-MCSI-GFP_dsRed; Amp^R^, Kan^R^	this study
pBR-GFP-[P*_comEA_*]dsRed-Kan	Upstream region of *comEA* (∼200 bp) cloned into *Stu*I site of pBR-GFP_dsRed_Kan; Amp^R^, Kan^R^	this study
pBR-[P*_pilA_*]GFP-[P*_comEA_*]dsRed-Kan	Upstream + part of coding region of *pilA* gene (∼600 bp) cloned into *Sma*I site of pBR-GFP-[P*_comEA_*]dsRed-Kan; Amp^R^, Kan^R^	this study
pBR-[P*_VC0047_*]GFP-[P*_comEA_*]dsRed-Kan	Upstream region of *VC0047* gene (∼500 bp) cloned into *Eco*RI/*Eco*RV site of pBR-GFP-[P*_comEA_*]dsRed-Kan; Amp^R^, Kan^R^	this study
pBR-[P*_hapA_*]GFP-[P*_comEA_*]dsRed-Kan	Upstream region of *hapA* gene (∼200 bp) cloned into *Eco*RI/*Xma*I site of pBR-GFP-[P*_comEA_*]dsRed-Kan; Amp^R^, Kan^R^	this study
pBR-[P*_gyrA_*]GFP-[P*_comEA_*]dsRed-Kan	Upstream region of *gyrA* gene (∼200 bp) cloned into *Eco*RI/*Xma*I site of pBR-GFP-[P*_comEA_*]dsRed-Kan; Amp^R^, Kan^R^	this study
pBR-[P*_recA_*]GFP-[P*_comEA_*]dsRed-Kan	Upstream region of *recA* gene (∼220 bp) cloned into *Eco*RI/*Xma*I site of pBR-GFP-[P*_comEA_*]dsRed-Kan; Amp^R^, Kan^R^	this study
pBR-[P*_clpX_*]GFP-[P*_comEA_*]dsRed-Kan	Upstream region of *clpX* gene (∼200 bp) cloned into *Eco*RI/*Xma*I site of pBR-GFP-[P*_comEA_*]dsRed-Kan; Amp^R^, Kan^R^	this study
pBR-[P*_ftsH_*]GFP-[P*_comEA_*]dsRed-Kan	Upstream region of *ftsH* gene (∼180 bp) cloned into *Eco*RI/*Xma*I site of pBR-GFP-[P*_comEA_*]dsRed-Kan; Amp^R^, Kan^R^	this study
pBR-[P*_comEC_*]GFP-[P*_comEA_*]dsRed-Kan	Upstream region of *comEC* gene (∼200 bp) cloned into *Eco*RI/*Xma*I site of pBR-GFP-[P*_comEA_*]dsRed-Kan; Amp^R^, Kan^R^	this study
pBR-[P*_comEA_*]GFP-[P*_pilA_*]dsRed-Kan	PCR product [P*_comEA_*]-[P*_pilA_*], using pBR-[P*_pilA_*]GFP-[P*_comEA_*]dsRED-Kan as template, cloned into *Xma*I/*Stu*I site of pBR-GFP_dsRed_Kan; Amp^R^, Kan^R^	this study
pBAD-*tfoX*-stop	*VC1153* (*tfoX*) in pBAD/Myc-HisA without tag; arabinose-inducible; Amp^R^	[22]
pUX-BF13	*ori*R6K, helper plasmid with Tn*7* transposition function; Amp^R^	[60]
pGP704::Tn7	pGP704 with mini-Tn7	Schoolnik lab collection; [37]
pGP704-mTn7-*araC*-*tfoX*	pGP704 with mini-Tn7 carrying *araC* and P*_BAD_*-driven *tfoX;* Amp^R^	this study

*: VC numbers according to [55].

**Table 2:** Natural transformation is dependent on cAMP, which is produced by adenylate cyclase (CyaA) and degraded by cAMP-degrading phosphodiesterase (CpdA).

*V. cholerae* strain	Competence induced#	Transformation frequency (±SD)
A1552-Tn*tfoX*	No	<d.l.
A1552-Tn*tfoX*	Yes	2.1×10^−4^ (±1.9×10^−4^)
ΔcyaA-Tn*tfoX*	No	<d.l.
ΔcyaA-Tn*tfoX*	Yes	<d.l.
ΔcpdA-Tn*tfoX*	No	<d.l.
ΔcpdA-Tn*tfoX*	Yes	7.1×10^−4^ (±4.7×10^−4^)*

# Competence was induced by the addition of 0.02% arabinose wherever indicated.

<d.l.: below detection limit;

*: Statistically significant difference in comparison to the wild type strain A1552-Tn*tfoX*; *P*<0.05; Average of at least three independent experiments.

### Quorum-sensing only regulates a subset of competence genes

**Figure 4:**
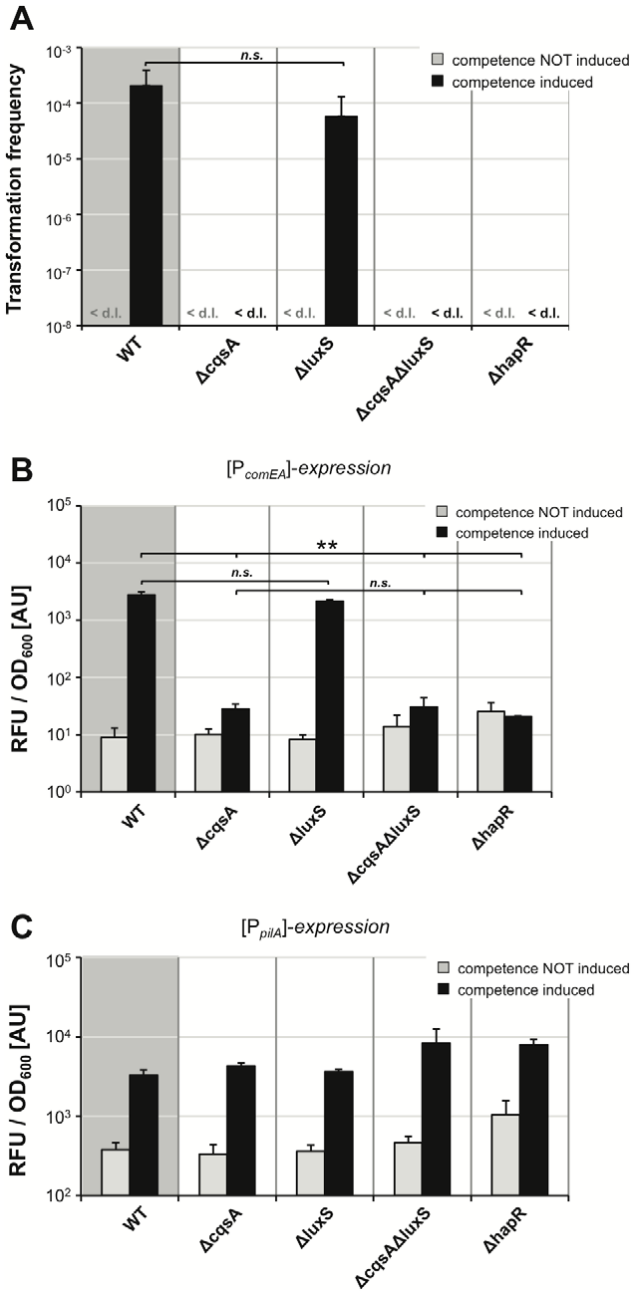
Quorum sensing only regulates a subset of competence genes. The *tfoX*-expression construct was transferred onto the chromosome of mutant *V. cholerae* strains, which were defective in the quorum-sensing circuit. Strains were grown in LB medium with or without 0.02% arabinose and tested for natural transformability (panel A), *comEA* (panel B), and *pilA* promoter-driven FP expression (panel C) as described for Figure 3. Experiments were repeated at least three times. Statistically significant differences were calculated using the Student's *t* test. * *P*<0.05, ** *P*<0.01, *n.s.* = not significant. <d.l. = below detection limit.

The next question we wanted to address was with respect to QS and the involved autoinducer molecules. We recently showed that the species-specific cholera autoinducer 1 (CAI-1; [46], [47]) plays a major role in natural competence for transformation and suggested that CAI-1 could be considered a competence pheromone [22]. We showed that the absence of the non-species-specific autoinducer 2 (AI-2; [48]) had no statistically significant effect on natural transformation on chitin surfaces, whereas strains devoid of CAI-1 synthesis were rarely transformable, and, even then, only at very low transformation frequencies [22]. However, as described above, chitin surfaces appear to be a rather heterogeneous environment, and cells might not all encounter the same autoinducer concentration in time and space, making chitin surface experiments difficult to conclusively judge the involvement of QS in the regulatory circuit of *V. cholerae*. Therefore, we investigated the role of QS in natural competence and transformation using our homogeneous competence-inducing system (Figure 4). We constructed Tn*tfoX*-containing *V. cholerae* deletion strains, which were devoid of either or both of the autoinducer-synthesizing enzymes CqsA and LuxS, or which lacked the gene encoding the major regulator of QS, HapR. These strains were grown in LB medium in the presence or absence of the competence-inducer arabinose and scored for natural transformability or competence gene promoter-driven FP expression (Figure 4). In the absence of inducer, the transformation frequency was consistently below the level of detection in all strains (Figure 4A). In the absence of the AI-2-producing enzyme LuxS, only a minor and statistically not significant decrease in transformation frequency upon competence induction was detectable when compared to the wild type parental strain (Figure 4A). More importantly, the dependency on CAI-1 was even enhanced when compared to our previous study on chitin surfaces, in that a deletion in the gene *cqsA* completely abolished natural transformation. This was also the case in strains devoid of both autoinducer synthases (CqsA and LuxS) and the major regulator of QS, HapR (Figure 4A). We argue that few occasionally detectable transformants in a CAI-1 negative mutant in our previous study on chitin flake surfaces [22] were the result of the heterogeneity of the chitin surface environment in which at least three components are not evenly distributed: autoinducers, transforming DNA and nuclease. However, under homogeneous conditions as tested here, a full dependency on CAI-1 is apparent.

We then visualized and quantified competence gene expression in these strains using the above-described FP report fusions (Figure 4B, 4C). The data for *comEA* promoter-driven FP expression (Figure 4B) mirrored the data of the transformation assay (Figure 4A). Under non-competence-inducing conditions, only the background fluorescence was measurable (in the range of the vector control shown in Figure 3). There was no statistically significant difference between the fluorescence signal detected in the wild type strain and the signal derived from the *luxS*-deficient strain upon competence-inducing conditions. A highly significant reduction of *comEA* promoter-driven FP gene expression was detected in the *cqsA*, *cqsA/luxS* and *hapR* negative strains. We also measured for the first time *pilA* promoter-driven FP expression in the different QS mutants and thus in the presence or absence of the two autoinducers. We observed that the expression pattern looked completely different from the *comEA* data; though the fluorescent signal increased upon competence induction, the fluorescence units were in the same range for all strains tested (Figure 4C). Therefore, we conclude that, in contrast to *comEA*, *pilA* is not subject to QS-dependent regulation.

Taken together, we provide evidence that CAI-1 is essential for *comEA* expression and natural transformation under homogeneous competence-inducing conditions. These data are in slight contrast to another study where a gradual decrease in *comEA* expression and natural transformation from a wild type *V. cholerae* strain towards an AI-2- and CAI-1-deficient strain, respectively, was displayed [21]. The authors of this study concluded that not only CAI-1 but also AI-2 contributes to natural transformation. We believe that the discrepancy between studies (the study described here, [21] and [22]) could reflect the different *V. cholerae* O1 El Tor strains used in both studies (A1552 here and in [22], versus C6707str in [21]). This hypothesis is in excellent agreement with a recent finding by Fong and Yildiz [36], who showed that the cAMP-CRP-mediated negative regulation of the biofilm regulatory gene *vpsR* only occurred in three out of four tested *V. cholerae* strains, namely strains A1552, N16961 and MO10. In contrast to this result, *V. cholerae* strain C6706 displayed no such regulation [36], highlighting the fact that different regulatory circuits exist in these *V. cholerae* strains.

### The amount of HapR within cells dictates *comEA* expression and nuclease repression

**Figure 5:**
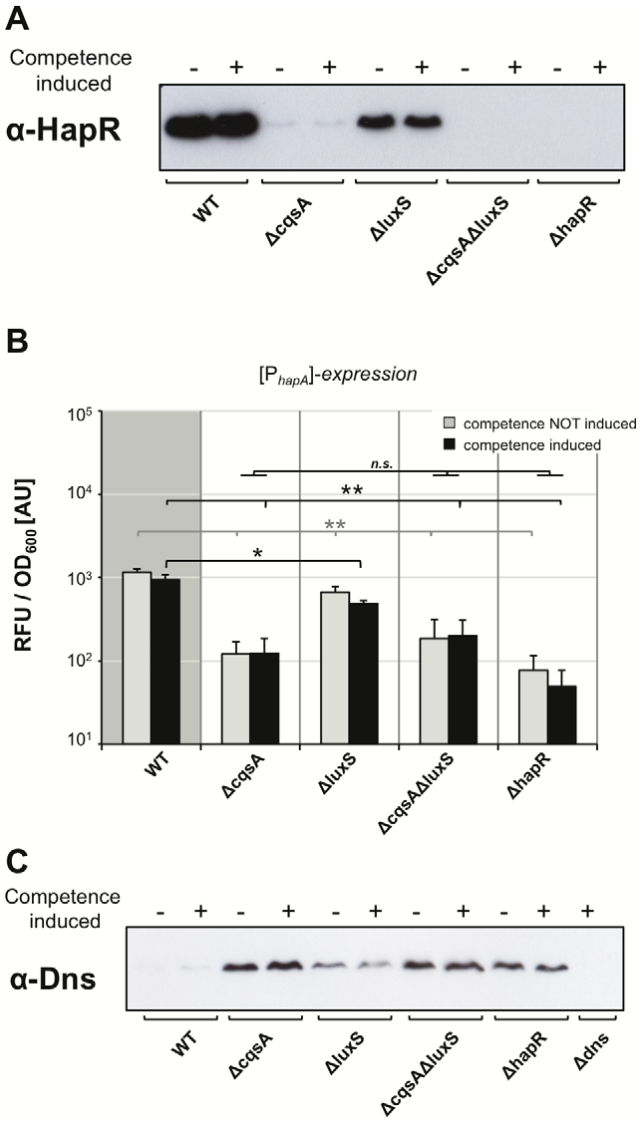
Correlation among HapR protein levels, *hapA* gene expression, and the nuclease Dns. Panel A and C: Proteins of the indicated strains, each containing artificially inducible *tfoX* on the chromosome, were separated by SDS-PAGE. After blotting, the relative abundance of proteins HapR (panel A) or Dns (panel C) were determined by detection with protein-specific antibodies. For each sample 6 µg (panel A) and 12 µg total protein (panel C), respectively, were applied per lane. Strains were tested under non-competence-inducing and competence-inducing conditions as indicated above each image. Panel B: HapR-dependent expression of *hapA* promoter-driven gene expression was quantified as described in Figure 3 for *comEA*/*pilA*. The growth conditions were as described for Figure 4. Averages of three independent experiments are indicated. Error bars indicate standard deviations. Statistically significant differences were calculated using the Student's *t* test. * *P*<0.05, ** *P*<0.01, *n.s.* = not significant.

The involvement of QS in natural transformation has previously been demonstrated by elucidating a role for HapR in the repression of a gene encoding the extracellular nuclease Dns [17]. This conclusion was mainly based on comparing a wild type *V. cholerae* strain to a ΔhapR mutant with respect to *dns* gene expression or nuclease activity. However, a direct correlation between HapR protein levels within the cell, *dns* repression, and *comEA* induction has never been demonstrated. We addressed this missing information by performing western blot analysis of non-competence-induced and competence-induced cells to detect the HapR protein within different QS mutant strains (Figure 5A). Whereas the amount of HapR did not differ between *tfoX*-induced and *tfoX*-uninduced cells significant differences between the tested strains were observable (Figure 5A). That is, whereas the HapR level was only slightly reduced in an AI-2-deficient strain (ΔluxS), HapR was almost undetectable in a strain lacking CAI-1 (ΔcqsA) (Figure 5A). Better detection was only possible upon overexposure of the film and such an overexposure did not reveal any HapR protein in the dual autoinducer mutant strain (ΔcqsAΔluxS) or the *hapR* negative control (Figure S7). This HapR protein pattern directly reflects the *comEA*-promoter driven FP expression data quantified in Figure 4B and also indicates that CAI-1 is the stronger autoinducer compared to AI-2 in *V. cholerae* strain A1552 (consistent with what was shown for strain C6707 using a heterologous read-out [49]). We also tested the expression of another QS-dependent but competence-independent gene, *hapA*, by transcriptionally fusing the *hapA* promoter to *gfp.* The *hapA* gene encodes a hemagglutinin protease (HA protease) and is positively regulated by HapR [50]. When we compared the *comEA* expression data (Figure 4B) to those of *hapA* (Figure 5B) a very similar expression pattern was observable under competence inducing conditions. We hypothesize that the low amount of HapR present in the *cqsA* mutant (Figure 5A and Figure S7) is not sufficient to activate expression of either *comEA* or *hapA*. A potential reason for this might be that HapR displays only a weak affinity for these promoters, which is consistent with a LuxR promoter affinity model described for *Vibrio harveyi* [51].

We also tested the impact of the HapR level on the protein amount of the nuclease Dns (Figure 5C) and observed an inverse correlation: Dns repression only occurred in those strains in which we detected high levels of HapR protein (e.g. WT and ΔluxS in Figure 5A). This is in good agreement with the absence of any transformants in a *cqsA* mutant (Figure 3A), as the abundance of the nuclease in this strain would avoid uptake of intact DNA. This is the first time that a direct correlation between HapR protein levels, nuclease levels and *comEA* expression has been shown, which is the critical link between QS and natural competence/transformation.

### Investigation of other *tfoX* and QS–regulated competence genes

**Figure 6:**
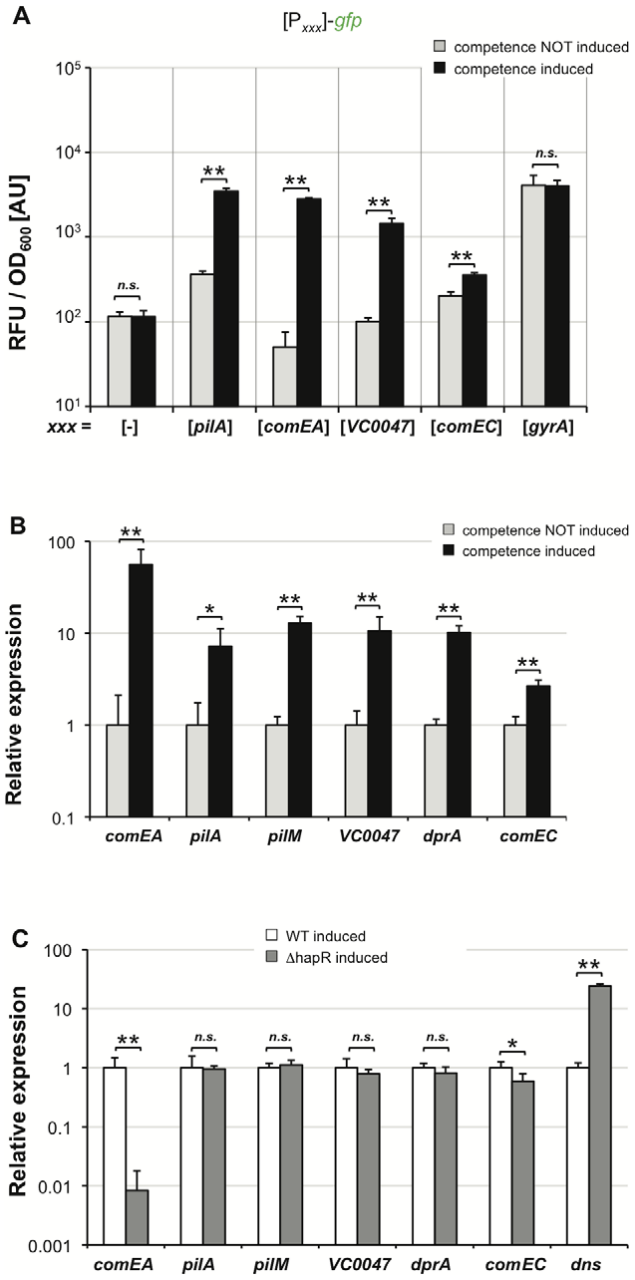
TfoX drives expression of QS–dependent and QS–independent competence genes. *V. cholerae* wild type strain containing artificially inducible *tfoX in cis* was tested for the expression of different competence genes. Panel A: Different transcriptional FP reporter fusions were tested for TfoX-dependent induction. These fusions were composed of the potential promoter region of the respective competence gene(s) (x-axis) fused to *gfp*. The housekeeping gene *gyrA* was tested as control. Relative fluorescence per OD_600_ unit is given on the y-axis. Panel B and C: qRT-PCR data comparing the relative expression of the indicated genes in a wild type strain under competence non-inducing and competence-inducing conditions (panel B). In panel C both the wild type strain and the hapR mutant were tested for competence gene expression under *tfoX*-expressing conditions. All panels depict averages of at least three independent experiments and error bars indicate standard deviations. Statistically significant differences were determined using Student's *t* tests. * *P*<0.05, ** *P*<0.01, *n.s.* = not significant.

Finally, we were curious to determine whether this chitin-independent, competence-inducing system would allow us to investigate other genes that are potentially involved in natural transformation. We initially focused on two potential promoter regions belonging to genes *VC0047* and *comEC*. The first promoter precedes *VC0047*, which is part of a four-gene operon (*VC0047-50*). The gene *VC0048* in this cluster encodes DprA, which is essential for natural transformation of *V. cholerae* [22]. The function of DprA in *Streptococcus pneumoniae* and probably also in *V. cholerae* is to protect the incoming single-stranded DNA from degradation and to convey the DNA to RecA-mediated recombination [52]. As shown in Figure 6A, a *tfoX*-dependent expression pattern was detectable in our *VC0047* promoter-driven *gfp* reporter strain.

Another gene that we have previously shown to be essential for transformation of *V. cholerae* is *comEC* ([22]; annotated as inner membrane transporter). As so far nothing was known about its regulation we sought to investigate whether *comEC* is regulated in a TfoX-dependent manner. Using a *comEC* promoter-driven *gfp* reporter strain we were able to measure a slight but statistically significant increase in *comEC* upon competence induction (Figure 6A). This is the first time that *comEC* has been shown to belong to the chitin-/TfoX-regulon in *V. cholerae* and as such being co-regulated with other competence genes.

We were also interested in the regulation of the *pilM-Q* operon as *pilQ* is also required for natural transformation [11], [22]. Thus, we fused the *pilM* promoter region to *gfp* and determined FP expression under competence inducing conditions. Unfortunately, the signal intensity was too low to allow us to unambiguously judge *pilM* promoter-driven expression. To overcome this obstacle we established quantitative RT-PCR in our laboratory to further monitor competence gene expression using our chitin-independent system. We first compared competence-uninduced to competence-induced cells with respect to expression of *comEA, pilA, pilM, VC0047, dprA and comEC*. As indicated in Figure 6B all of these genes were significantly induced upon competence-induction. This again confirmed the TfoX-dependent regulation of *comEC* shown in Figure 6A, which was missed in previous chitin/TfoX-dependent expression studies [11], [31]. We suggest that this was the case, as the change in expression did not pass the significance filter in these microarray expression studies. Indeed, the fold-difference for *comEC* expression upon competence induction was only 2.7 (Figure 6B). Interestingly, this 2.7-fold increase in *comEC* expression is in the same range as what we observed using the *comEC* FP reporter construct (1.8-fold change; Figure 6A), though the latter system was plasmid-based.

**Figure 7:**
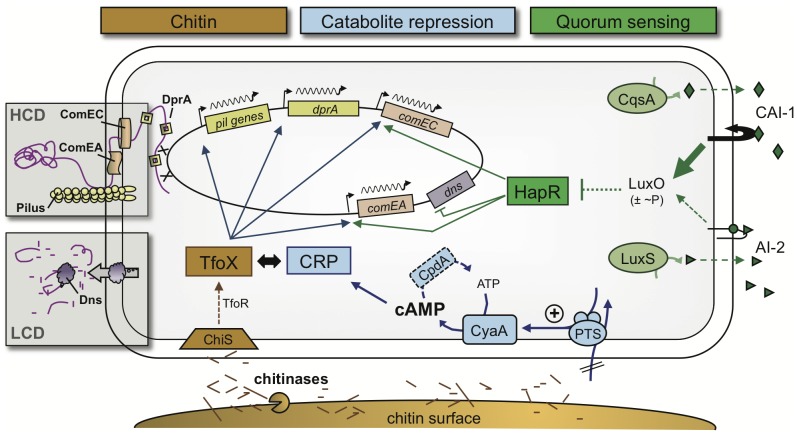
Model of the regulatory network of natural competence and transformation of *V. cholerae*. At least three extracellular and intracellular signaling molecules must be present to allow natural transformation to occur in *V. cholerae*. 1) Chitin degradation products such as chitin oligomers, which lead to the induction of the sRNA TfoR and the main regulator of transformation TfoX (chitin pathway shown in brown). 2) The secondary messenger cAMP, which has to accumulate within cells (CCR pathway shown in blue). 3) Extracellular autoinducers, with an emphasis on the stronger autoinducer CAI-1, which feed into the quorum-sensing circuit (shown in green). Whereas chitin- and TfoX-dependent induction and the requirement for cAMP and CRP are universal for all, so far investigated, competence genes, the QS-dependent circuit regulates only a subset of those, such as *comEA* and *comEC*. Therefore, QS acts as a switch in gene expression and is responsible for the final fate of the surrounding DNA (boxed areas). At a low cell density (LCD), the DNA (shown in purple) is degraded by the nuclease Dns. As a consequence, the cells are non-transformable. At a high cell density (HCD) and, therefore, high abundance of the autoinducer CAI-1, the nuclease gene *dns* is transcriptionally repressed, whereas *comEA* and *comEC* are activated. ComEA as well as ComEC then contribute to the DNA uptake process, probably due to their ability to shuffle the DNA through the periplasmic space and the inner membrane, respectively.

Finally, we wanted to test whether any of these other competence genes is also regulated in a QS-dependent manner. We therefore tested and compared the expression of these genes under competence-inducing conditions in a wild type and *hapR* negative strain, respectively (Figure 6C). Apart from *comEA* and *dns,* only *comEC* turned out as also HapR-dependent (Figure 6C, *P* = 0.0157). This is in nice agreement with our regulatory model in which the fate of the surrounding DNA is determined by QS (Figure 7 and conclusion below).

### Concluding remarks and proposed model of the regulatory network

In this study, we analyzed the regulatory network of chitin-induced natural competence and transformation in *V. cholerae* in its full complexity (Figure 7). Our results suggest that under homogeneous conditions bistability is unlikely for *V. cholerae*. However, the conditions might be less homogeneous in time and space around biotic surfaces (e.g., different concentrations of autoinducers and PTS sugars, which interfere with natural competence via QS and CCR, respectively). Such “environmental heterogeneity” might foster a non-synchronized response by the chitin-associated bacteria. We hypothesize that due to such a heterogeneity competence gene expression appeared absent in a subpopulation of bacteria grown on chitin beads, whereas housekeeping genes were almost uniformly expressed throughout the population (Figure 1 and Figure S1).

Based on the results obtained in this study and combined with the knowledge from earlier studies by us and others [11], [17], [22], [23], we developed a model for the regulatory network of natural competence and transformation (Figure 7). The model predicts the interplay between three pathways for the initiation of competence: chitin sensing followed by TfoX activation, CCR, and QS. The first pathway, the dependency on a chitin surface or on chitin oligomers (e.g., GlcNAc_2–6_) for competence induction, was discovered as these compounds lead to an upregulation of potential competence genes. Within these competence genes were the so-called *pil* genes, which encode for a type IV pilus that is potentially involved in the DNA uptake process [22], [31] (Figure 7). Chitin also led to an induction of the gene encoding the main regulator of natural transformation, TfoX [11], [31]. This finding is supported by recent studies that show that chitin oligomers (GlcNAc_>2_) lead to an increase of *tfoX* transcription but also to its enhanced translation [18]. The latter effect could be explained after the discovery of a chitin-induced small RNA TfoR, which activates translation of TfoX mRNA and, therefore, contributes to the induction of natural competence [20]. Attempts from our group to look at transcriptional reporter fusions between *tfoX* and *gfp* were unsuccessful, which was most likely due to the low activity of the *tfoX* promoter (M. Lo Scrudato, M. Grasser, M. Blokesch, unpublished). The flow of information in this part of the regulatory circuit (e.g., chitin sensing) is therefore as follows (Figure 7): the presence of chitin is sensed by the chitin sensor ChiS, due to chitinase-released GlcNAc di-/oligomers [31], [53], and the signal is then transferred via TfoR towards the production of TfoX.

As this chitin-dependent pathway is well established, we excluded it in the second part of this study and designed a chitin-independent, competence-inducing system, which is based on artifical *tfoX* expression (though not overproduction). With this chitin-independent system, we obtained transformants at comparable frequencies to our optimized chitin-induced transformation protocol [19] and at 10- to 10,000-fold higher frequencies than recent studies by other groups [18], [20], [21]. As the induction occurs under homogeneous conditions in this system, it allowed us to us to better investigate the two other pathways involved in natural competence regulation, CCR and QS.

Based on experimental data showing that glucose interferes with chitin-induced transformation, Meibom *et al*. suggested that catabolite repression might be involved in the competence phenotype [11]. This hypothesis was recently extended as we showed that competing PTS-dependent carbon sources indeed repress natural transformation [23]. Such sugars are known to play a role in the intracellular accumulation of the secondary messenger cAMP, which, together with the cAMP receptor protein CRP, contributes to chitin surface colonization, chitin degradation and natural competence [23]. In this current study, we circumvented the problem that CCR mutants are often impaired for colonization and growth on chitin as a sole carbon source [23] by uncoupling natural competence-induction from chitin. This allowed us to better understand the dependency on cAMP for competence gene expression. Indeed we observed a change in natural transformability upon creating an imbalance in the intracellular cAMP pool, either by inhibiting cAMP production or, alternatively, by avoiding cAMP degradation. The latter effect was accomplished by deleting the gene encoding the cAMP phosphodiesterase, an enzyme that has never been studied in *V. cholerae* before. We also confirmed that the TfoX-induced expression of *pilA* and *comEA* requires cAMP, which is consistent with the idea proposed, but not yet unequivocally demonstrated, for *Haemophilus influenzae* that TfoX and cAMP-CRP act in concert to induce competence genes [40], [41], [54] (Figure 7).

The third pathway that participates in natural competence induction is quorum-sensing (QS). The involvement of QS in competence initiation was initially speculated based on different facts. First, the first sequenced strain of *V. cholerae* N16961 [55] was non-transformable [11]. In this strain, the indigenous *hapR* gene contains a frameshift mutation that renders it non-functional [56]. This deficiency could be overcome by providing a functional copy of *hapR* back in *cis* [11]. The second line of evidence for the involvement of QS in natural competence and transformation came from the fact that cells were more efficiently transformable after longer growth on chitin surfaces, which is equivalent to higher cell densities [11]. However, such a finding could also be explained by elevated intracellular cAMP levels after extended growth on chitin. The involvement of QS in natural transformation was more directly demonstrated by elucidating a role for HapR in the repression of a gene encoding the extracellular nuclease Dns [17]. The finding that HapR “acts as a negative regulator for *dns* transcription” was recently also confirmed by others [57]. The study by Blokesch and Schoolnik unambiguously demonstrated that the HapR-induced repression of the nuclease is the main, but not the only, contribution of QS to natural transformability, and the authors proposed that HapR also acts as a positive regulator of *comEA* [17]. This speculation was based on earlier microarray expression data, which showed that *comEA* expression upon growth on chitin was significantly reduced in the absence of HapR [11]. A QS-dependent regulation of *comEA* has recently been demonstrated [21] but differed from the data presented here as discussed above. In the current study, we extended this analysis by studying natural transformation and competence gene expression using the same competence-inducing conditions. Furthermore, we simultaneously monitored the expression of two competence genes, namely *comEA* and *pilA*, and compared their expression levels in different QS mutants. Based on the data provided, we conclude that *comEA* but not *pilA* is regulated by QS (Figure 7). We also showed for the first time a direct link between intracellular HapR protein concentrations, nuclease repression and *comEA* induction (Figure 4 and Figure 5).

Finally, we tested other competence genes, namely *pilM*, *VC0047*, *dprA*, and *comEC*, with respect to their expression levels; for these genes less or no information concerning their regulation was known before this study. We conclusively showed that all tested competence genes were dependent on induction by TfoX but only a part of them was also co-regulated in a QS-dependent manner (Figure 6). In fact only three genes showed a significantly different expression under competence inducing conditions in a wild type strain compared to a *hapR* mutant, namely *dns*, *comEA* and *comEC* (Figure 7). The encoded proteins are all directly involved in the fate of the surrounding DNA: whereas Dns degrades free DNA at low cell density, ComEA and ComEC are required for the DNA uptake process once high cell density is reached on the chitin surface (Figure 7). Further studies will follow to provide a better insight into the DNA uptake process itself.

## Materials and Methods

### Bacterial strains and plasmids

Bacterial strains and plasmids used in this study are listed in Table 1. *E. coli* strains DH5α [58] and One Shot PIR1 or PIR2 (Invitrogen) were used as hosts for cloning purposes. *E. coli* strain S17-1λpir [59] served as mating donor for plasmid transfers between *E. coli* and *V. cholerae*.

### Media and growth conditions

Overnight cultures were grown in LB medium under aerobic conditions. Defined artificial seawater medium (DASW) [11] supplemented with vitamins (MEM, Gibco) and 0.1% casamino acids (Becton, Dickson and Company) was used for static growth of *V. cholerae* on chitin beads (New England Biolabs) or for growth under shaking conditions with N-acetylglucosamine (GlcNAc) or hexa-N-acetylchitohexaose (GlcNAc)_6_ (obtained from Seikagaku Corporation via Northstar BioProducts and LuBioScience, Lucerne, Switzerland) as sole carbon source. Thiosulfate Citrate Bile Salts Sucrose (TCBS) agar plates were prepared following the manufacturer's instructions (Fluka) and were used to counter-select *E. coli* strains after triparental mating with *V. cholerae*. Experiments for artificial *tfoX* expression were performed in LB medium with or without the addition of 0.02% arabinose. LB medium and LB agar plates were supplemented with antibiotics wherever required. The final concentrations of antibiotics were 75 µg/ml for kanamycin, 50 or 100 µg/ml for ampicillin and 50 µg/ml for gentamicin. *V. cholerae* cells were always grown at 30°C.

### Construction of *V. cholerae* strains

The *V. cholerae cpdA* deletion strain was constructed using plasmid pGP704-28-SacB-ΔcpdA (Table 1) and the gene disruption method described previously [31]. The oligonucleotides used for the construction of the deletion plasmid are indicated in Table S1.

*V. cholerae* strains carrying artificially inducible *tfoX* on the chromosome (e.g., in *cis*) were created by triparental mating between the respective *V. cholerae* strain (Table 1), *E. coli* strain S17λpir/pUX-BF13 (providing the transposase function; [60]), or *E. coli* strain S17λpir/pGP704-mTn7-*araC*-*tfoX.* The latter plasmid consists of the suicide vector pGP704 as the backbone and the mini-Tn7 transposon [43] containing the gene cluster *araC-*P*_BAD_*-*tfoX* as cargo. This gene cluster was amplified by PCR from the plasmid pBAD-*tfoX*-stop [22] (primers indicated in Table S1).

### Construction of transcriptional reporter fusions

The FP reporter constructs are all based on plasmid pBR322 [61]. Initially, this plasmid was modified by (partial) deletion of the tetracycline resistance cassette as well as the constitutive promoter P*_Tet_*_,_ resulting in plasmid pBR-Tet_MCSI (Table 1). A kanamycin resistance cassette (*aph*) as well as promoter-less versions of the genes *gfp* and *dsRed* (DsRed.T3[DNT]), both pointing in the opposite direction, were inserted into pBR-Tet_MCSI to yield plasmid pBR-GFP_dsRed_Kan. Details of all plasmids are included in Table 1. The primers used for construction of the plasmids are listed in Table S1 (synthesized by Microsynth, Switzerland). PCR mixtures, PCR programs, restriction enzyme digestions, primer phosphorylation, and ligations followed standard protocols recommended by the manufacturers of the enzymes (Roche, Switzerland and New England Biolabs via Bioconcept, Switzerland).

### Growth of *V. cholerae* on chitin beads

*V. cholerae* strains were grown aerobically in LB medium until an OD_600_ of ∼0.3. Cells were harvested, washed in DASW medium and mixed with an equal volume of prewashed chitin beads (New England Biolabs) and three volumes of DASW (final volume 1 ml). The mixture was supplemented with vitamins, 0.1% casamino acids, and kanamycin (for plasmid maintenance). The bacteria were grown as standing cultures in 12-well plates. Bacteria were visualized by epifluorescence microscopy after 24 and 48 hours of growth, respectively.

### Epifluorescence microscopy settings

Specifications of the epifluorescence microscope were as follows: Zeiss Axio Imager M2 microscope; Zeiss High Resolution Microscopy Camera AxioCam MRm; Illuminator HXP 120 as fluorescence light source (metal halide); objective used in this study: Plan-Apochromat 100×/1.40 Oil Ph3 M27 (WD = 0.17 mm). Filters relevant to this study were Zeiss Filter set 63 HE mRFP shift free; EX BP 572/25, BS FT 590, EM BP 629/62 and Zeiss filter set 38 Endow GFP shift free; EX BP 470/40, BS FT 495, EM BP 525/50. Image acquisition was done using the Zeiss AxioVision software. Images were rotated, cropped and uniformly enhanced with respect to contrast and brightness using Zeiss AxioVision and Adobe Photoshop CS3.

Microscopy image analysis was performed using the Matlab-based MicrobeTracker Suite [62] and according to the instructions given by the inventors (http://microbetracker.org/). Fluorescence intensities were normalized with respect to the area of the cell and the exposure time.

### Induction of natural competence by hexa-N-acetylchitohexaose and flow cytometry

Chitin-dependent but surface-independent growth of *V. cholerae* was performed as previously described [23] using 2 mM of hexa-N-acetylchitohexaose GlcNAc_6_ (obtained from Seikagaku Corporation via Northstar BioProducts and LuBioScience, Lucerne, Switzerland) as sole carbon source. The same strains were grown in parallel with N-acetylglucosamine (GlcNAc; control). Bacteria were harvested at an OD_600_ of 0.8. Bacteria were either immediately visualized by epifluorescence microscopy or fixed in 2% paraformaldehyde for 30 min. Fixed samples were washed, diluted in PBS (1∶5), and analyzed by flow cytometry using a BD LSR II Flow cytometer. BD FACSDiva software was used for data acquisition. GFP signals were excited with a blue laser (488 nm) and detected with a 525/50 filter. DsRed.T3[DNT] was excited with a green laser (561 nm) and detected with a 585/15 filter. For each sample, 100,000 events were counted in total. Biologically independent experimental replicates (three for Figure 1 and Figure 2; two for Figure S1 and Figure S3) were performed within two weeks. One representative experiment is depicted in Figure 2 and the averages of the mean fluorescence intensities from three different biological replicates are shown in Figure S4.

### Chitin-independent induction of competence

Strains used for chitin-independent competence-induction all carried inducible *tfoX* (*araC*-P*_BAD_*_-_ *tfoX*) on a mini-Tn7 transposon [43] within the chromosome. Induction was accomplished by growth in LB supplemented with 0.02% arabinose.

For transformation assays, cells were grown until an OD_600_∼1.0. At that point, aliquots of 0.5 ml cultures were transferred to 1.5-ml tubes and supplemented with 2 µg/ml transforming DNA (gDNA of strain A1552-LacZ-Kan; [19]). Tubes were shaken horizontally for 5 h. Transformed cells and total colony forming units (CFU) were enumerated by a variation of a previously described method [63]. Briefly, the cultures underwent a serial dilution, and 5 µl of each dilution step was spotted in duplicate or triplicate on plain LB or LB containing 75 µg/ml kanamycin plates, respectively. Transformation frequencies were calculated as number of transformants divided by total number of CFUs. Each experiment was repeated at least three independent times, and the averages of all experiments are given in the figures (± standard deviations). Statistical analyses of transformation frequencies were performed on log-transformed data [64] using a two-tailed Student's *t* test.

Strains were grown in LB medium with or without 0.02% arabinose to investigate transcriptional FP reporter fusions. After 24 h, the cells were either visualized by epifluorescence microscopy or measured for relative fluorescence using a Tecan Infinite M200 plate reader. Parameters for detection of GFP were: excitation (Ex) at 485 nm (9 nm bandwidth) and emission (Em) at 515 nm (20 nm bandwidth). DsRed.T3[DNT] was detected using Ex 560 (9)/Em 587 (20) nm, as previously described for DsRed.T3 [28]. The samples were also measured with respect to their OD_600_, and results are given as relative fluorescence units (RFU) divided by OD_600_ values. The averages of three biological replicates are shown. Error bars indicate standard deviations. Statistically significant differences were analyzed using two-tailed Student's *t* tests.

### Quantitative reverse transcription PCR (qRT-PCR)

*V. cholerae* strains were grown in 10 ml LB in the absence or presence of 0.02% arabinose until they reached an optical density of ∼1.7. At that time 5 ml of each culture was harvested and lysed in 1 ml Tri Reagent (Sigma). The samples were stored at −80°C. RNA isolation, DNase treatment, and reverse transcription using 1 µg of total RNA as template was done as previously described [23]. The obtained cDNA was diluted 40-fold and served as template in the qPCR. The primers used for the qPCR are indicated in Table S1. The qPCR mix was based on the *Fast Start Essential DNA Green Master Mix* (Roche, Switzerland), a ready-to use hot start reaction mix optimized for qPCR using the Light Cycler Nano system from Roche. The qPCR mix further contained 0.5 µM of each primer. The qPCR run using the Light Cycler Nano was performed according to these parameters: a denaturation step at 95**°**C for 10 min followed by 40 cycles of 95**°**C for 10 s, 60**°**C for 20 s, 72**°**C for 20 s. Each run was finished with a melting-curve ranging from 50**°**C to 95**°**C to validate specific amplification, which was also initially confirmed for each primer pair by standard PCR and visualization of PCR fragments in agarose gels. For each sample a reverse transcriptase-negative control was also performed while doing the reverse transcription and the respective samples were analyzed with at least three independent primer pairs to exclude residual DNA contaminations. A standard curve was prepared for each primer pair using purified genomic DNA of *V. cholerae* A1552 diluted in PCR grade water (from 1000 to 0.1 pg gDNA template). A negative control lacking any template was also tested for each primer pair. The expression values were normalized against expression of the housekeeping gene *gyrA* as previously described [65]. However, as discussed above the expression of *gyrA* might be dependent on DNA supercoiling and the cell cycle as shown for other bacteria [34], [35]. We therefore compared the relative expression of the four housekeeping genes *gyrA*, *recA*, *clpX*, *ftsH* as well as *comEA* in two different *V. cholerae* strains (WT and ΔhapR). The expression patterns were extremely similar no matter whether we normalized the expression data against *gyrA* expression (Figure S8A) or against *recA* expression (Figure S8B) as internal controls. The results were analyzed using the Light Cycler Nano software.

### Preparation of cell lysates and determination of total protein concentration

For the preparation of cell lysates, bacteria were grown aerobically in LB medium in the absence or presence of 0.02% arabinose. Cells were harvested after reaching an OD_600_ of ∼1.5, resuspended in SDS-loading buffer (2×Laemmli buffer without β-mercaptoethanol and bromophenol blue), and boiled at 98°C for 15 min. Total protein concentration was quantified using the Pierce BCA Protein Assay kit (Thermo Scientific) before the addition of β-mercaptoethanol and bromophenol blue (7.5% and 0.01% final concentrations, respectively).

### Generation of antibodies against HapR and Dns

Antibodies raised against the peptides derived from the proteins HapR, Dns, and TfoX were produced by Biomatik (Canada). The polyclonal antibody production service included suggestions for the design of two peptides per protein, peptide synthesis, conjugation of the peptides to the carrier proteins (keyhole limpet hemocyanin), and immunization of two rabbits per peptide mix. Polyclonal antibodies were affinity purified against the antigen and checked by ELISA. Each antibody was first validated for potential cross-reactions at the same size as the target protein using western blot analysis of the respective know-out strains.

### Electrophoretic separations and Western blotting

Separation of proteins under denaturing conditions was conducted by SDS-PAGE using 15% acrylamide gels [66], [67]. The amount of total protein loaded per lane was 6 µg, 12 µg, and 50 µg for HapR, Dns, and TfoX detection, respectively. For western blot analysis, the proteins were transferred onto PVDF western blotting membranes (Roche), stained with amido black to verify transfer efficiency, incubated in blocking buffer, and reacted with primary antibodies directed against HapR (1∶5000), Dns (1∶1000), or TfoX (1∶2000). Detection of the primary antibody was performed using a secondary goat anti-rabbit IgG antibody conjugated to peroxidase (Sigma A9169; used at a 1∶20,000 dilution). Signals were revealed using Lumi-Light^PLUS^ Western Blotting substrate (Roche, Switzerland) and were recorded by exposure to chemiluminescence-detecting films (Amersham Hyperfilm ECL, GE Healthcare).

## Supporting Information

Figure S1 Visualization of housekeeping compared to competence gene expression on chitin surfaces. *V. cholerae* cells were grown on chitin beads and were visualized as described for Figure 1. The diverse transcriptional FP reporter tested were: I: the vector control containing promoter-less *gfp* and *dsRed*; II to V: a reporter constructs containing *gfp* driven by the *gyrA* promoter, the *recA* promoter, the *clpX* promoter and the *ftsH* promoter, respectively, oppositely oriented to the *comEA* promoter-driven *dsRed* gene. Bacteria were grown statically for 24 h before pictures were taken. The order of the images is the same as for Figure 1. Scale bar = 5 µm.
(TIF)

Figure S2 Homogeneous expression of competence genes under homogeneous competence-inducing conditions. *V. cholerae* strains were grown in the presence of either GlcNAc or (GlcNAc)_6_ as described for Figure 2. Competence gene expression was visualized using epifluorescence microscopy. Images in the upper row show the pictures taken in the green (left) and red (right) channel. The image on the lower left shows a merged image of the phase contrast picture and the images of both fluorescence channels. The signals in the original images were also quantified using MicrobeTracker [62]. Thus, graphs below microscopy images show histograms of the fluorescent intensities (log-transformed; including a two-period moving average trendline); the plot in the lower right shows the correlation of the bacteria with respect to both FPs (given as log scale). Reporter fusions are indicated above each panel. Panel A&B: promoter-less *gfp* and *dsRed* reporter; panel C&D: [P*_pilA_*]-*gfp*/[P*_comEA_*]-*dsRed*; and panel E&F: [P*_comEA_*]-*gfp* and [P*_pilA_*]-*dsRed*. Panel G&H: [P*_gyrA_*]-*gfp*/[P*_comEA_*]-*dsRed*. Scale bar in all images = 5 µm.
(PDF)

Figure S3 Housekeeping genes are expressed in the majority of cells under homogeneous conditions. The indicated bacterial reporter strains were grown aerobically in DASW medium with GlcNAc_6_ as inducer of competence. Promoter-driven FP gene expression was either visualized by epifluorescence microscopy (images) or quantified for fluorescence intensities using flow cytometry (graphs below the fluorescence images). Scale bar in all images = 5 µm.
(PDF)

Figure S4 Reproducibility of mean fluorescence intensities as quantified by flow cytometry. The mean fluorescence intensity for promoter-driven *gfp* expression (panel A) and the corresponding *dsRed* expression (panel B) was measured in the three biological replicates of the experiment corresponding to Figure 2. Average values are indicated for competence non-inducing and competence-inducing conditions and errors bars represent the standard deviations.
(TIF)

Figure S5 Artifical *tfoX* induction in *cis* does not lead to TfoX overproduction. 50 µg of total protein derived from the two indicated strains, which were grown in the presence of the indicated concentration of L-arabinose, were separated by SDS-PAGE. After blotting, the abundance of the TfoX protein was determined with protein-specific antibodies. The position of TfoX is indicated on the right. The upper image was obtained after 10 min of film exposure. For the lower image the film was exposed for 60 min. The white arrow at the bottom indicates the conditions used in earlier studies, whereas the gray arrows reflects the chromosomally encoded inducible but not overproducing *tfoX* system described in this study.
(TIF)

Figure S6 TfoX-dependent competence induction requires cAMP within the cells. Wild type *V. cholerae* cells or derivatives of the parental strain lacking adenylate cyclase (CyaA) or cAMP phosphodiesterase (CpdA) harboring *cis*-encoded *tfoX* were grown in rich medium in the absence (gray bars) or presence of the inducer arabinose (black bars). Bacteria were scored for *comEA*- (panel A) and *pilA-* (panel B) driven expression using a 96-well plate reader. The relative fluorescence units were normalized to the OD_600_ values. Average are from three independent replicates. <d.l. = below detection level.
(TIF)

Figure S7 Detection of HapR within different QS mutant strains. Proteins of the indicated strains, each containing artificially inducible *tfoX* on the chromosome, were separated by SDS-PAGE. After blotting, the relative abundance of the HapR protein was determined by detection with protein-specific antibodies. For each sample, 6 µg of total protein was applied per lane. Strains were tested under non-competence-inducing and competence-inducing conditions as indicated above the figure. The image represents an overexposed film (in comparison to Figure 5A) to detect weaker signals.
(TIF)

Figure S8 Normalization of qRT-PCR data using either *gyrA* or *recA* as internal controls results in comparable expression patterns. qRT-PCR data comparing the relative expression of the genes *gyrA/recA* (lane 2 in panel A and B, respectively), *clpX* (lane 3), *ftsH* (lane 4), and the competence gene *comEA* (lane 5) in a ΔhapR-Tn*tfoX* strain compared to the normalized expression in the wild type strain A1552-Tn*tfoX* (lane 1). Both strains were grown under competence inducing conditions. Both panels show averages of three independent biological replicates and error bars indicate standard deviations.
(TIF)

Table S1 Primers used in this study.
(DOCX)
