# Supplementary figures and images for: The Regulatory Network of Natural Competence and Transformation of Vibrio cholerae
Source: PLoS Genet. 2012 Jun 21;8(6):e1002778. doi: 10.1371/journal.pgen.1002778 (PMC3380833; doi:10.1371/journal.pgen.1002778)

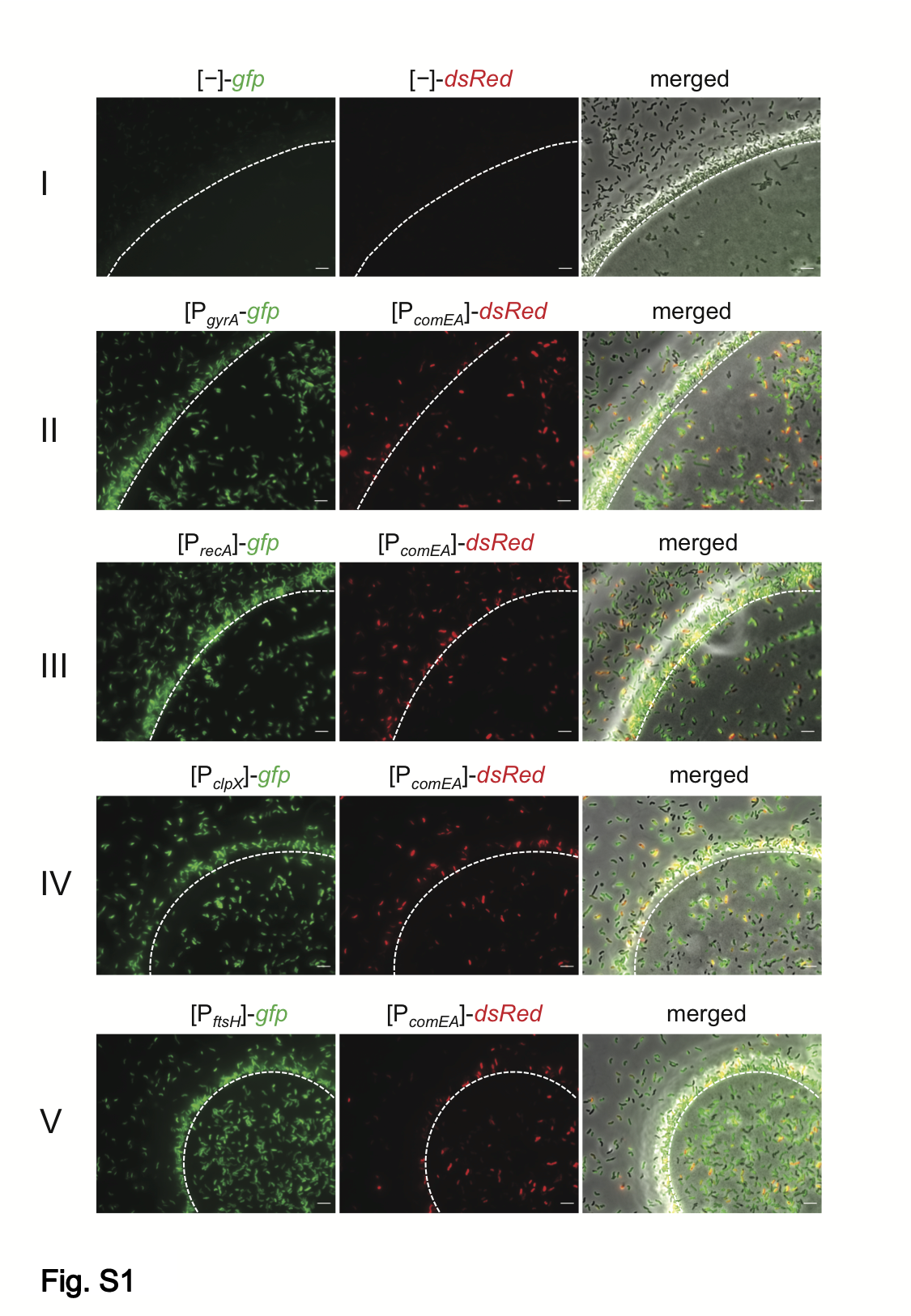

Supplement: Figure S1 — Visualization of housekeeping compared to competence gene expression on chitin surfaces. V. cholerae cells were grown on chitin beads and were visualized as described for Figure 1. The diverse transcriptional FP reporter tested were: I: the vector control containing promoter-less gfp and dsRed; II to V: a reporter constructs containing gfp driven by the gyrA promoter, the recA promoter, the clpX promoter and the ftsH promoter, respectively, oppositely oriented to the comEA promoter-driven dsRed gene. Bacteria were grown statically for 24 h before pictures were taken. The order of the images is the same as for Figure 1. Scale bar = 5 µm. (TIF) [file pgen.1002778.s001.tif]

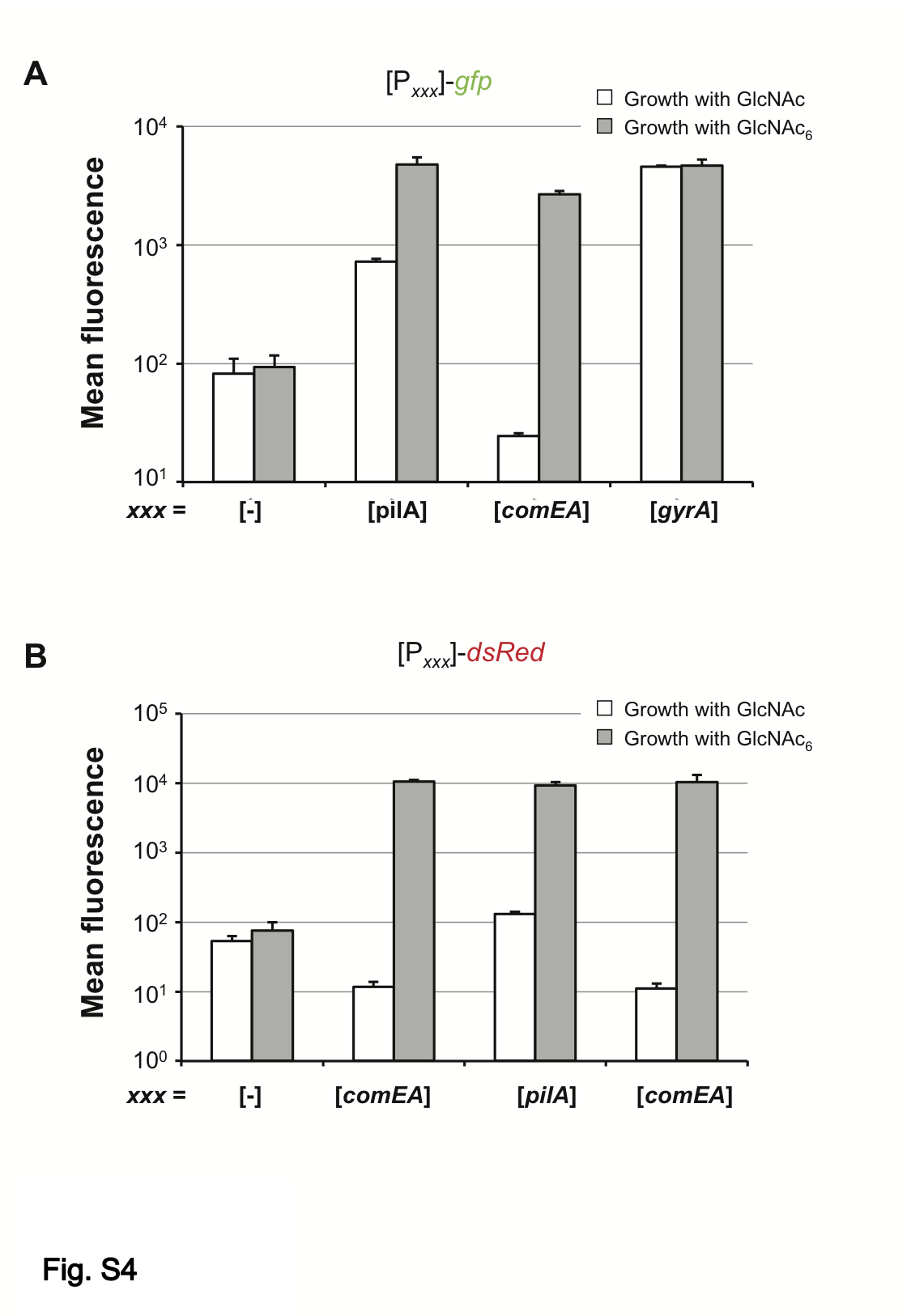

Supplement: Figure S4 — Reproducibility of mean fluorescence intensities as quantified by flow cytometry. The mean fluorescence intensity for promoter-driven gfp expression (panel A) and the corresponding dsRed expression (panel B) was measured in the three biological replicates of the experiment corresponding to Figure 2. Average values are indicated for competence non-inducing and competence-inducing conditions and errors bars represent the standard deviations. (TIF) [file pgen.1002778.s004.tif]

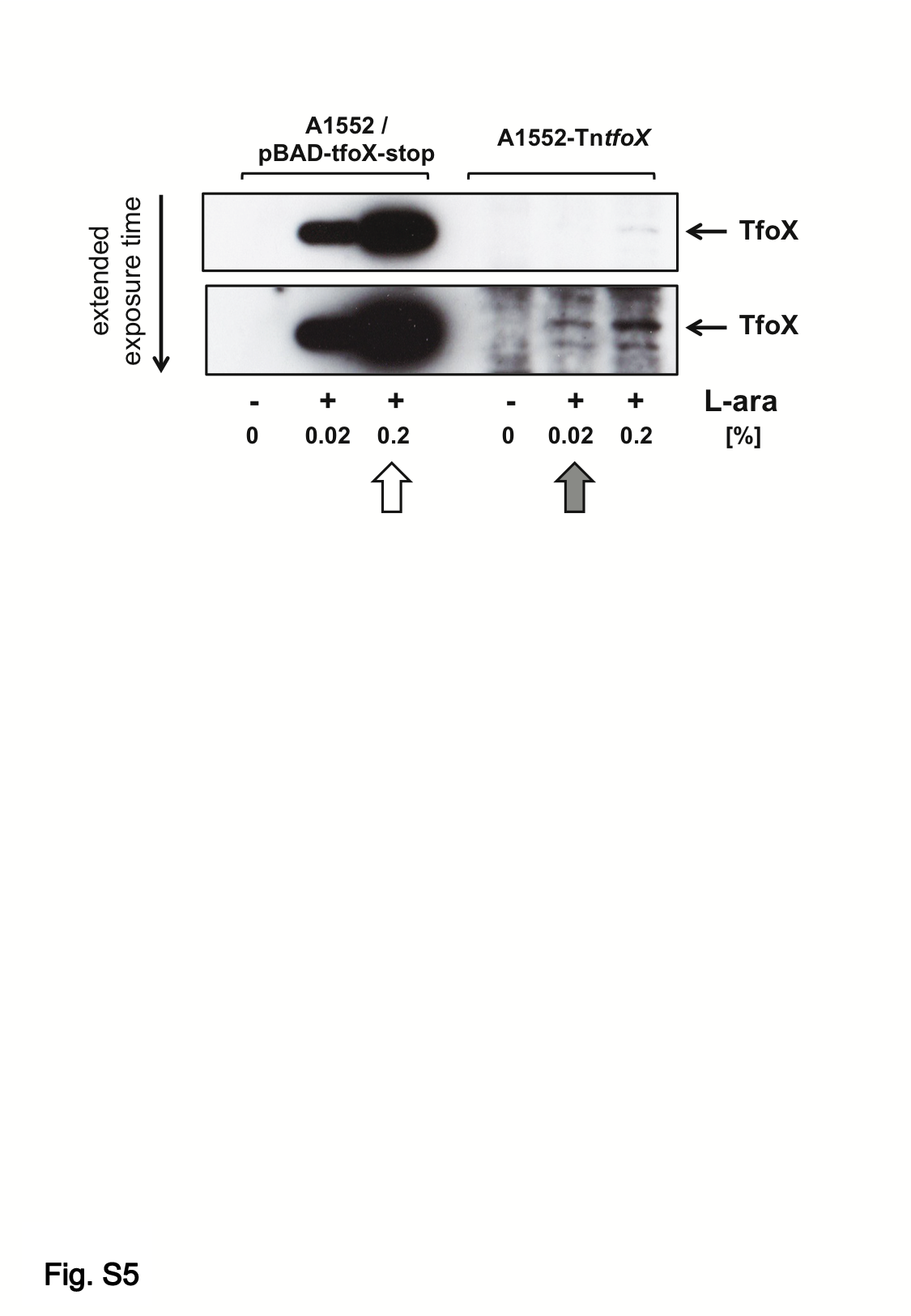

Supplement: Figure S5 — Artifical tfoX induction in cis does not lead to TfoX overproduction. 50 µg of total protein derived from the two indicated strains, which were grown in the presence of the indicated concentration of L-arabinose, were separated by SDS-PAGE. After blotting, the abundance of the TfoX protein was determined with protein-specific antibodies. The position of TfoX is indicated on the right. The upper image was obtained after 10 min of film exposure. For the lower image the film was exposed for 60 min. The white arrow at the bottom indicates the conditions used in earlier studies, whereas the gray arrows reflects the chromosomally encoded inducible but not overproducing tfoX system described in this study. (TIF) [file pgen.1002778.s005.tif]

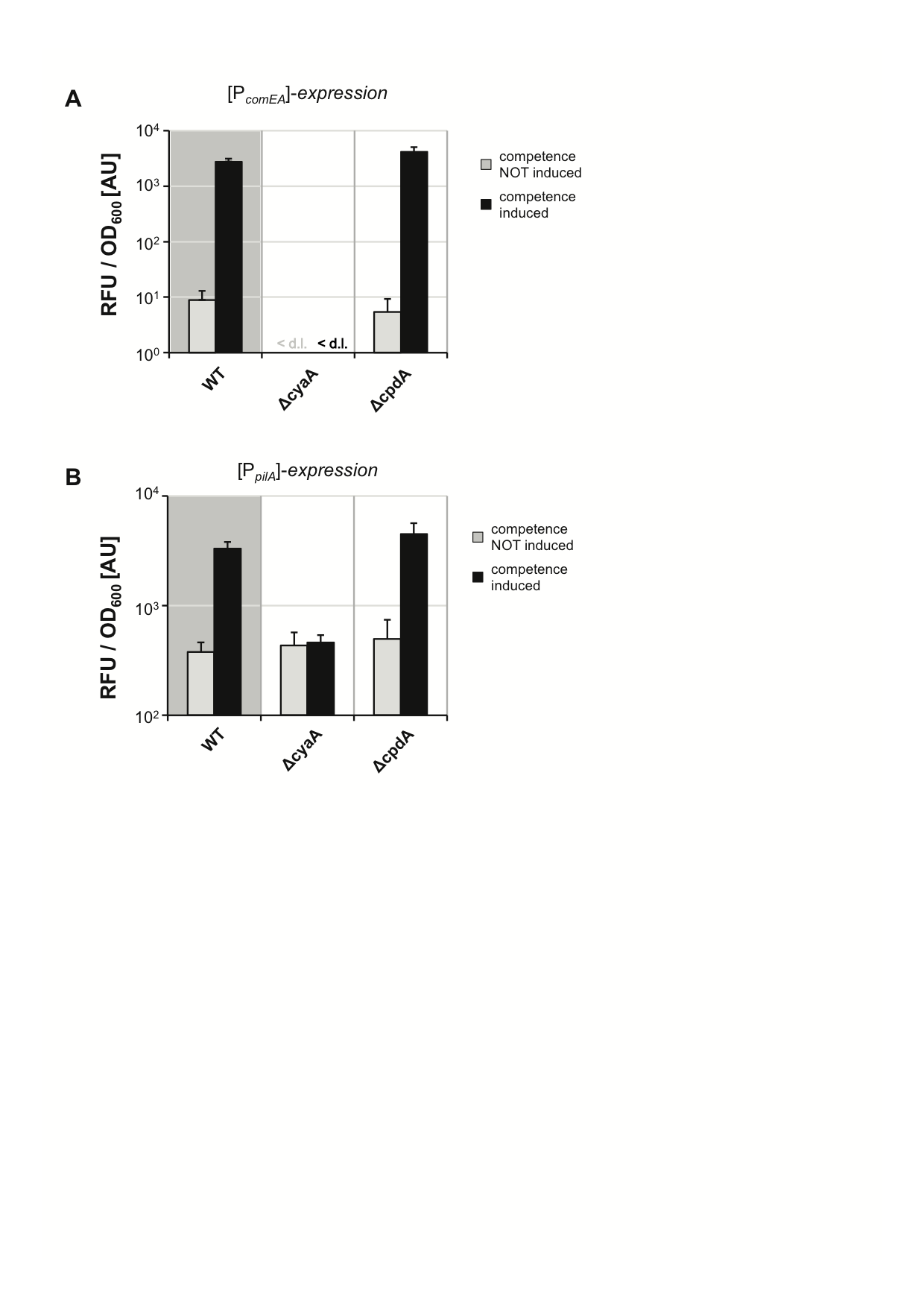

Supplement: Figure S6 — TfoX-dependent competence induction requires cAMP within the cells. Wild type V. cholerae cells or derivatives of the parental strain lacking adenylate cyclase (CyaA) or cAMP phosphodiesterase (CpdA) harboring cis-encoded tfoX were grown in rich medium in the absence (gray bars) or presence of the inducer arabinose (black bars). Bacteria were scored for comEA- (panel A) and pilA- (panel B) driven expression using a 96-well plate reader. The relative fluorescence units were normalized to the OD600 values. Average are from three independent replicates. <d.l. = below detection level. (TIF) [file pgen.1002778.s006.tif]

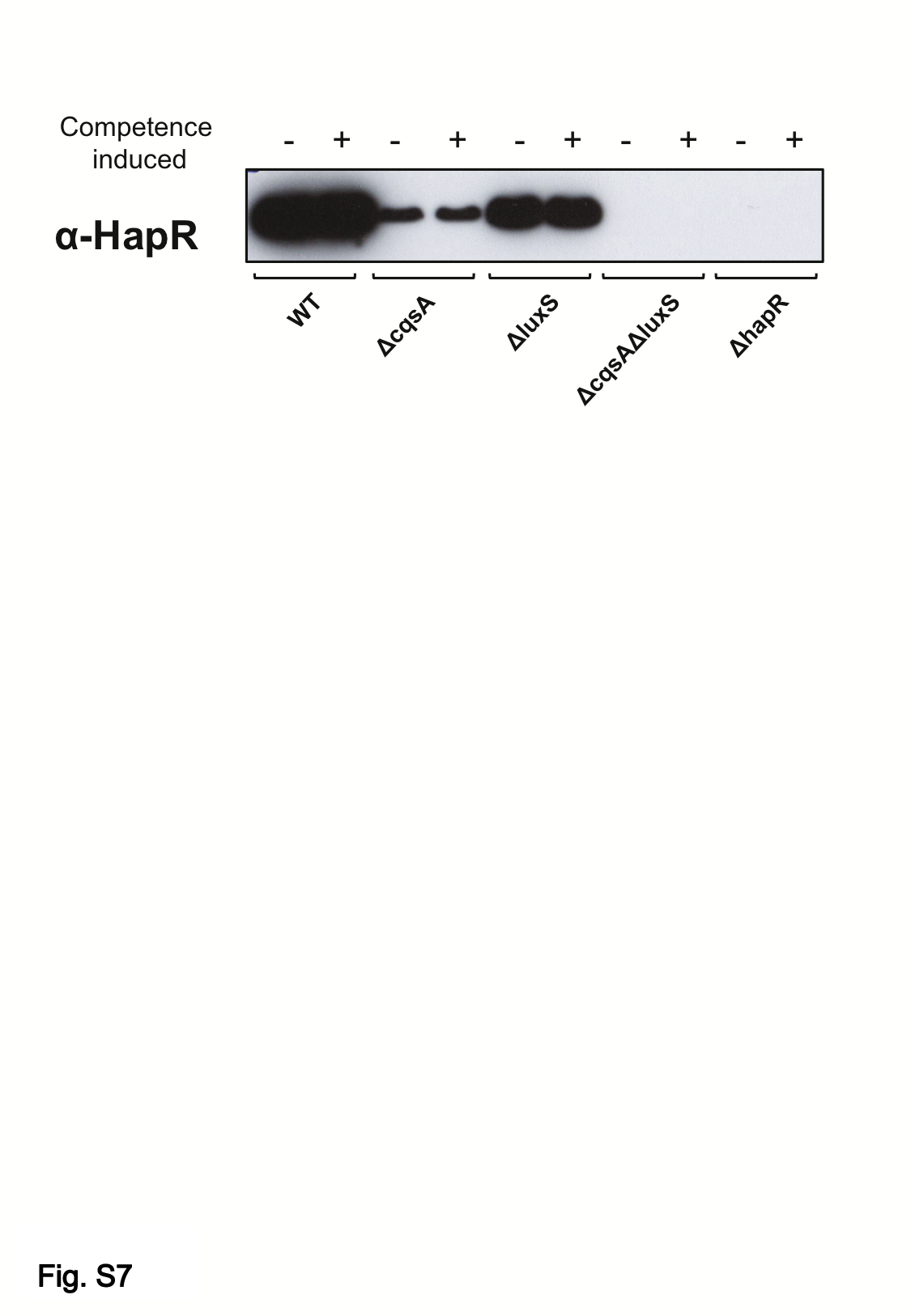

Supplement: Figure S7 — Detection of HapR within different QS mutant strains. Proteins of the indicated strains, each containing artificially inducible tfoX on the chromosome, were separated by SDS-PAGE. After blotting, the relative abundance of the HapR protein was determined by detection with protein-specific antibodies. For each sample, 6 µg of total protein was applied per lane. Strains were tested under non-competence-inducing and competence-inducing conditions as indicated above the figure. The image represents an overexposed film (in comparison to Figure 5A) to detect weaker signals. (TIF) [file pgen.1002778.s007.tif]

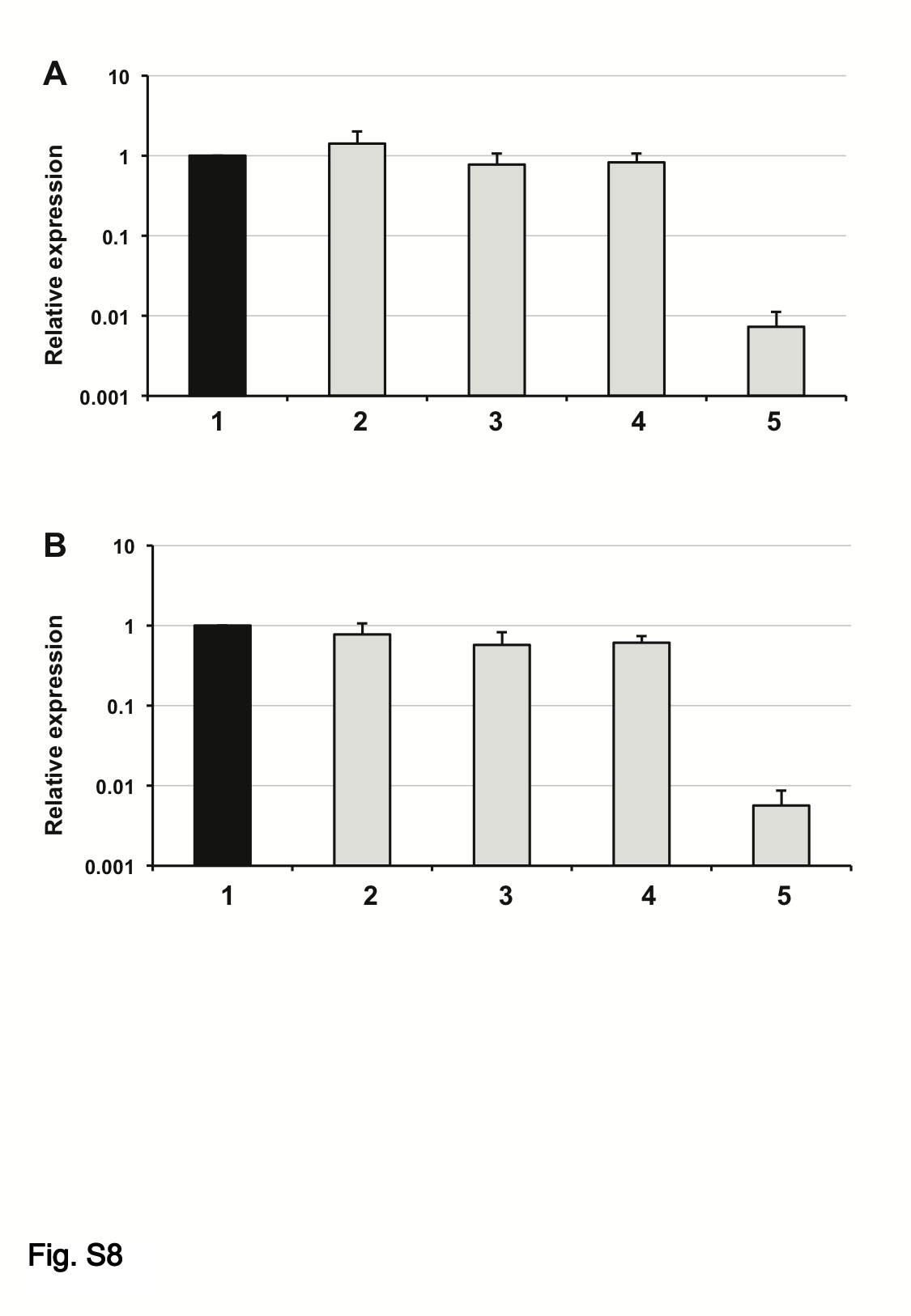

Supplement: Figure S8 — Normalization of qRT-PCR data using either gyrA or recA as internal controls results in comparable expression patterns. qRT-PCR data comparing the relative expression of the genes gyrA/recA (lane 2 in panel A and B, respectively), clpX (lane 3), ftsH (lane 4), and the competence gene comEA (lane 5) in a ΔhapR-TntfoX strain compared to the normalized expression in the wild type strain A1552-TntfoX (lane 1). Both strains were grown under competence inducing conditions. Both panels show averages of three independent biological replicates and error bars indicate standard deviations. (TIF) [file pgen.1002778.s008.tif]
