# Supplementary material for: The Regulatory Network of Natural Competence and Transformation of Vibrio cholerae
Source: PLoS Genet. 2012 Jun 21;8(6):e1002778. doi: 10.1371/journal.pgen.1002778 (PMC3380833; doi:10.1371/journal.pgen.1002778)
Supplement: Figure S2 — Homogeneous expression of competence genes under homogeneous competence-inducing conditions. V. cholerae strains were grown in the presence of either GlcNAc or (GlcNAc)6 as described for Figure 2. Competence gene expression was visualized using epifluorescence microscopy. Images in the upper row show the pictures taken in the green (left) and red (right) channel. The image on the lower left shows a merged image of the phase contrast picture and the images of both fluorescence channels. The signals in the original images were also quantified using MicrobeTracker [62]. Thus, graphs below microscopy images show histograms of the fluorescent intensities (log-transformed; including a two-period moving average trendline); the plot in the lower right shows the correlation of the bacteria with respect to both FPs (given as log scale). Reporter fusions are indicated above each panel. Panel A&B: promoter-less gfp and dsRed reporter; panel C&D: [PpilA]-gfp/[PcomEA]-dsRed; and panel E&F: [PcomEA]-gfp and [PpilA]-dsRed. Panel G&H: [PgyrA]-gfp/[PcomEA]-dsRed. Scale bar in all images = 5 µm. (PDF) [file pgen.1002778.s002.pdf]

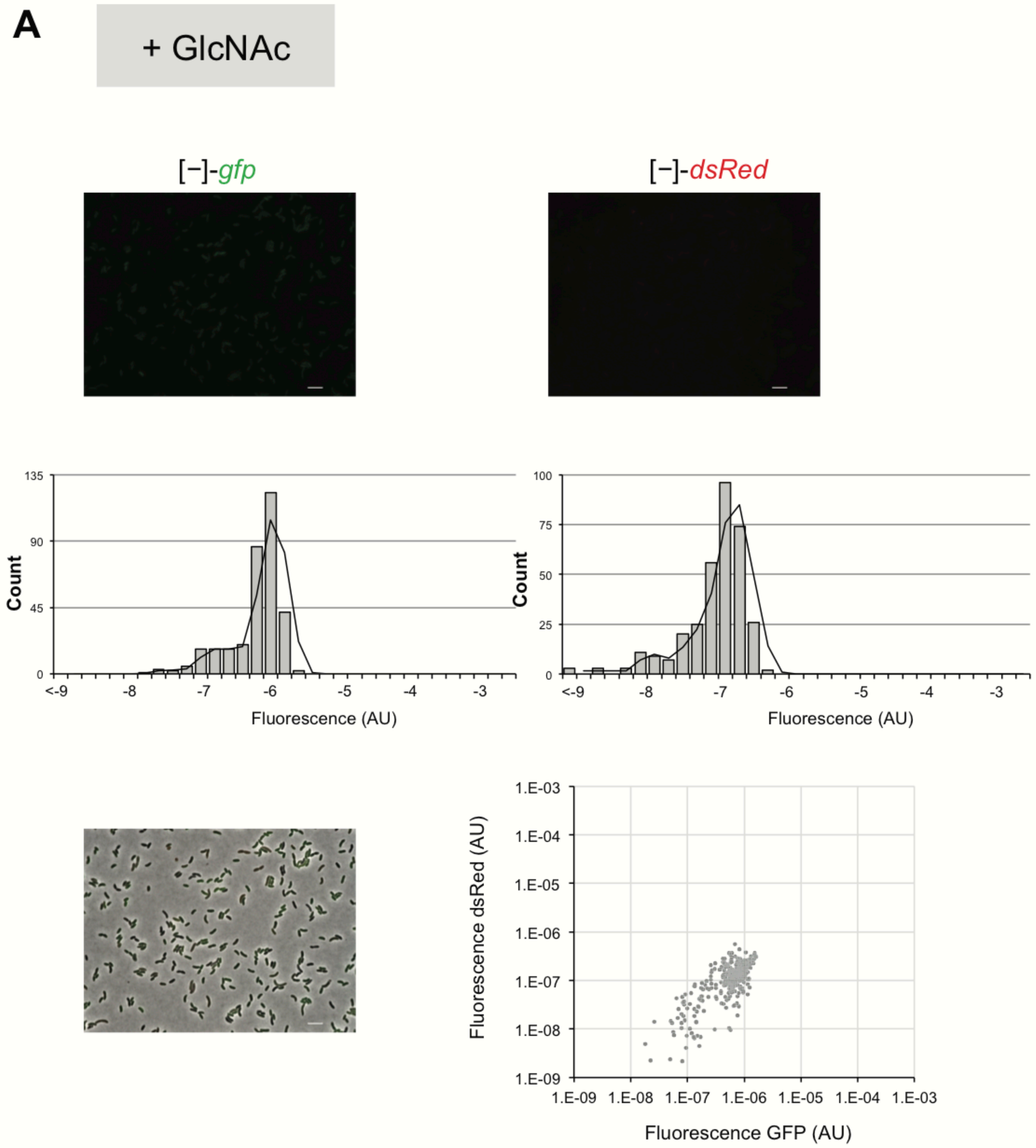
A
+ GlcNAc
[−]-gfp
[−]-dsRed
135
90
45
0
Count
<-9
-8
-7
-6
-5
-4
-3
Fluorescence (AU)
100
75
50
25
0
Count
<-9
-8
-7
-6
-5
-4
-3
Fluorescence (AU)
1.E-03
1.E-04
1.E-05
1.E-06
1.E-07
1.E-08
1.E-09
Fluorescence dsRed (AU)
1.E-09 1.E-08 1.E-07 1.E-06 1.E-05 1.E-04 1.E-03
Fluorescence GFP (AU)

Fig. S2A

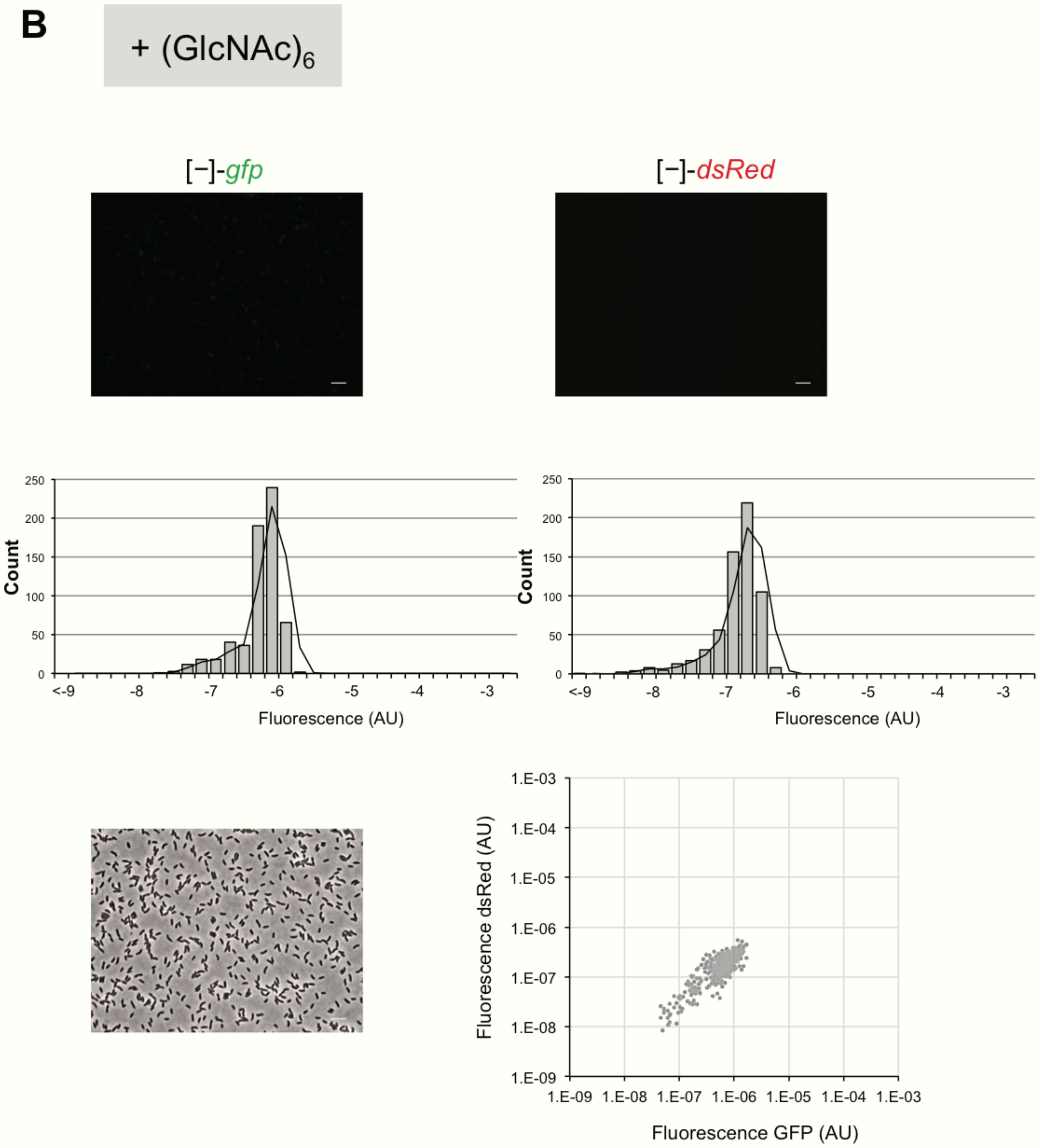
B
+ (GlcNAc)6
[−]-gfp
[−]-dsRed
250
200
150
100
50
0
Count
<-9
-8
-7
-6
-5
-4
-3
Fluorescence (AU)
1.E-03
1.E-04
1.E-05
1.E-06
1.E-07
1.E-08
1.E-09
Fluorescence dsRed (AU)
Fluorescence GFP (AU)

Fig. S2B

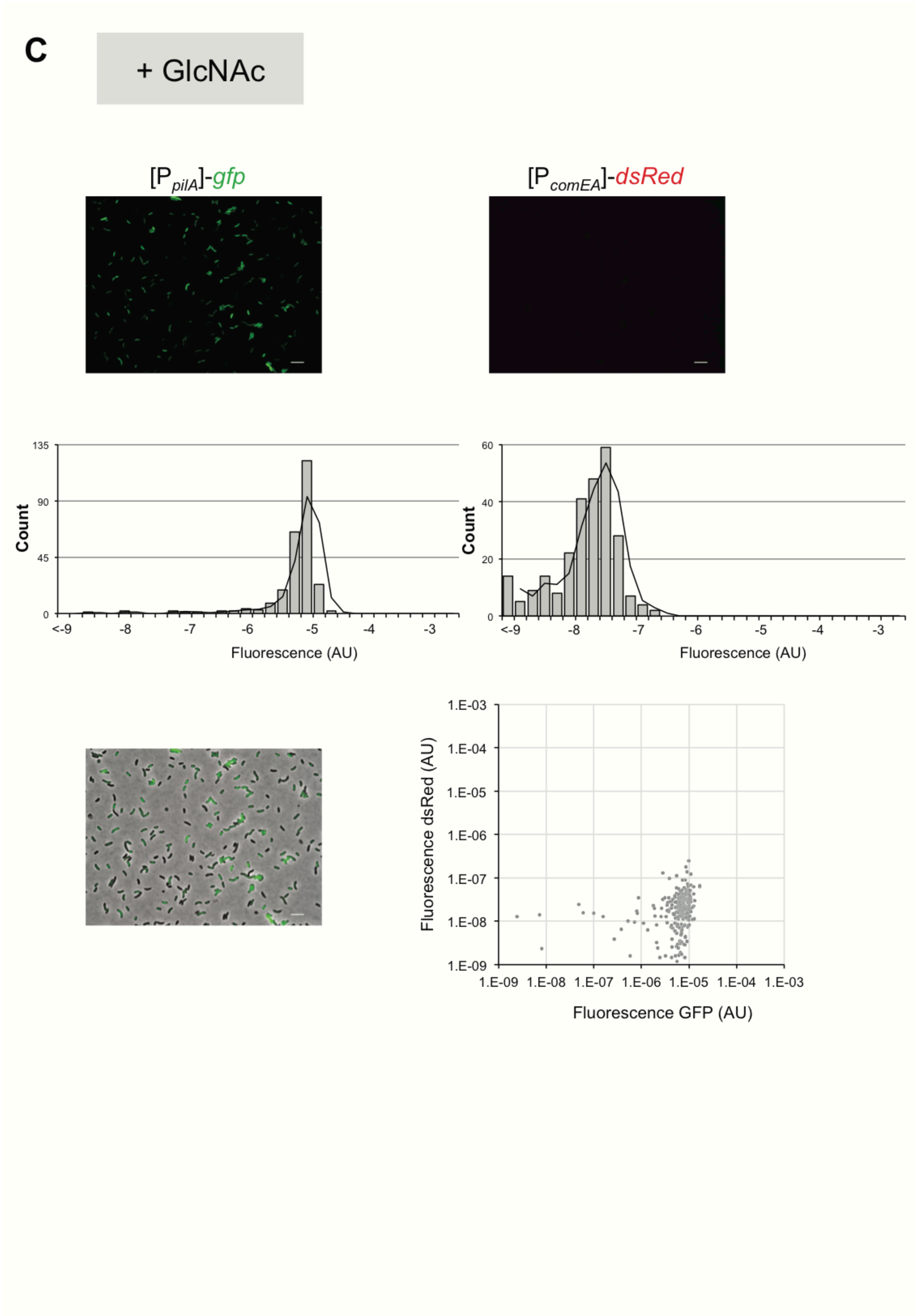
C
+ GlcNAc
[P pilA]-gfp
[P comEA]-dsRed
Count
Fluorescence (AU)
Fluorescence dsRed (AU)
Fluorescence GFP (AU)

Fig. S2C

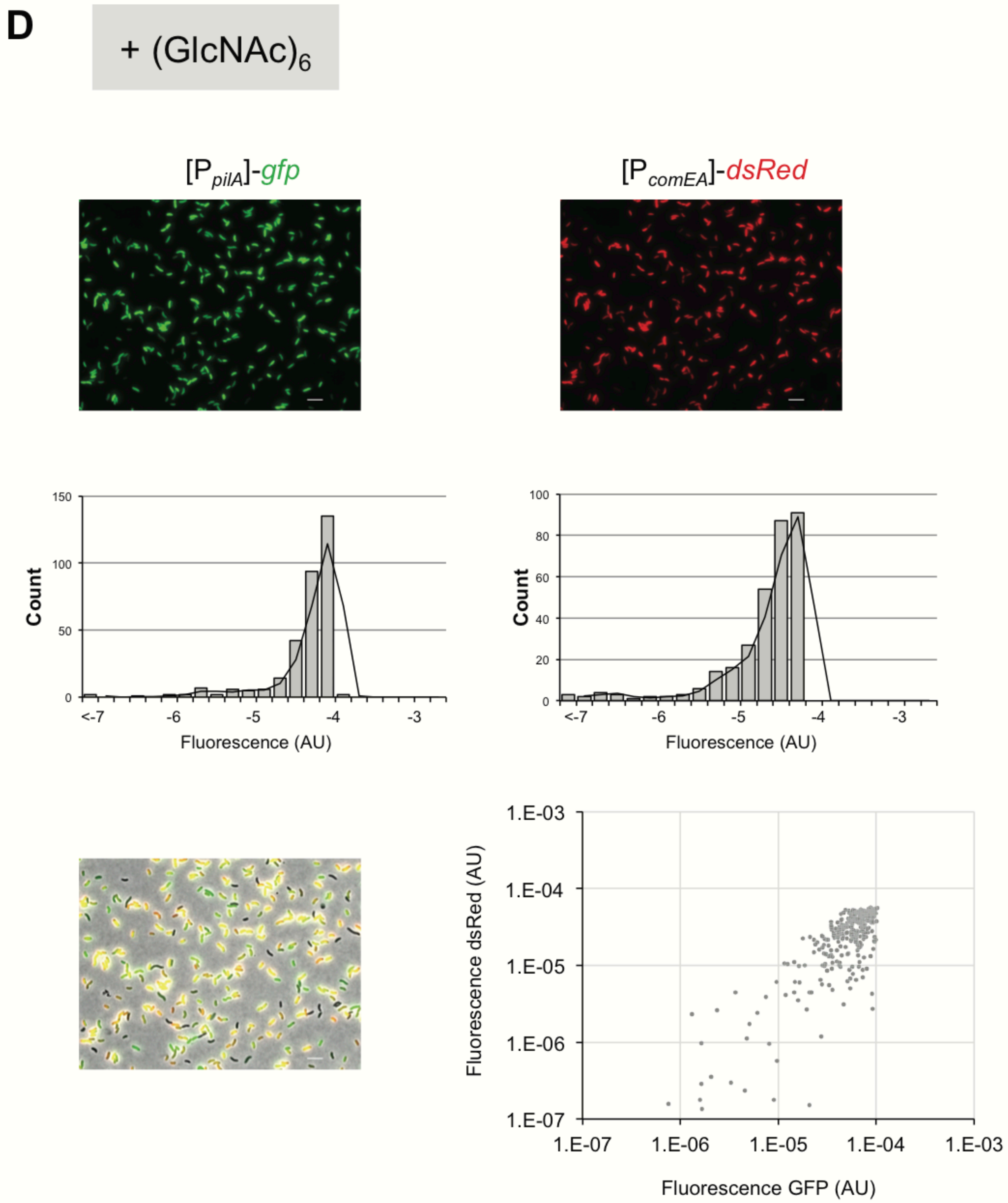

D
+ (GlcNAc)6
[PpilA]-gfp
[PcomEA]-dsRed
Count
Fluorescence (AU)
150
100
50
0
<-7
-6
-5
-4
-3
80
60
40
20
Fluorescence dsRed (AU)
Fluorescence GFP (AU)
1.E-03
1.E-04
1.E-05
1.E-06
1.E-07

Fig. S2D

E

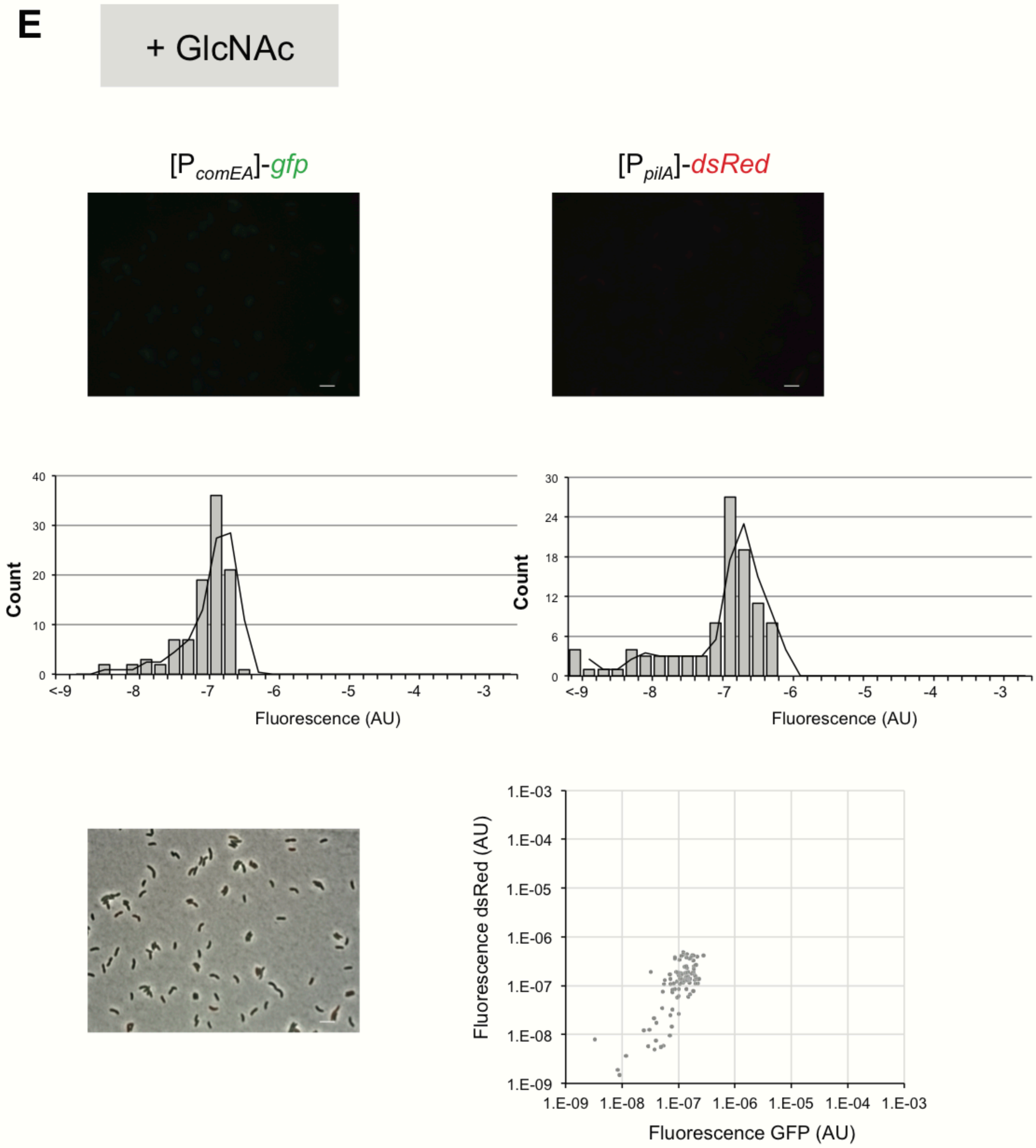
+ GlcNAc
[P comEA]-gfp
[P pilA]-dsRed
Count
Fluorescence (AU)
Fluorescence dsRed (AU)
Fluorescence GFP (AU)
1.E-09
1.E-08
1.E-07
1.E-06
1.E-05
1.E-04
1.E-03

Fig. S2E

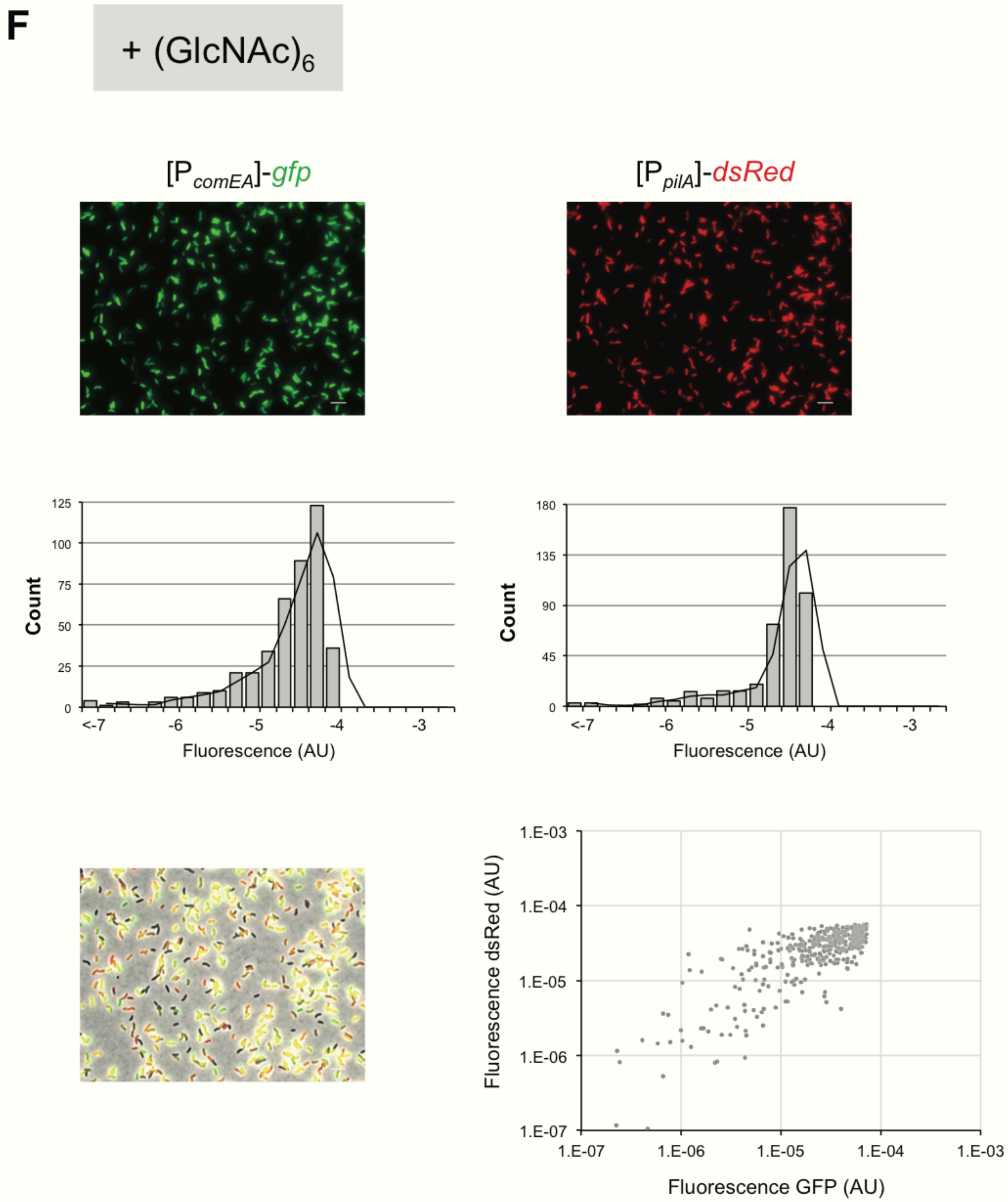
F
+ (GlcNAc)6
[PcomEA]-gfp
[PpilA]-dsRed
Count
Fluorescence (AU)
125
100
75
50
25
0
<-7
-6
-5
-4
-3
180
135
90
45
Fluorescence dsRed (AU)
Fluorescence GFP (AU)
1.E-03
1.E-04
1.E-05
1.E-06
1.E-07

Fig. S2F

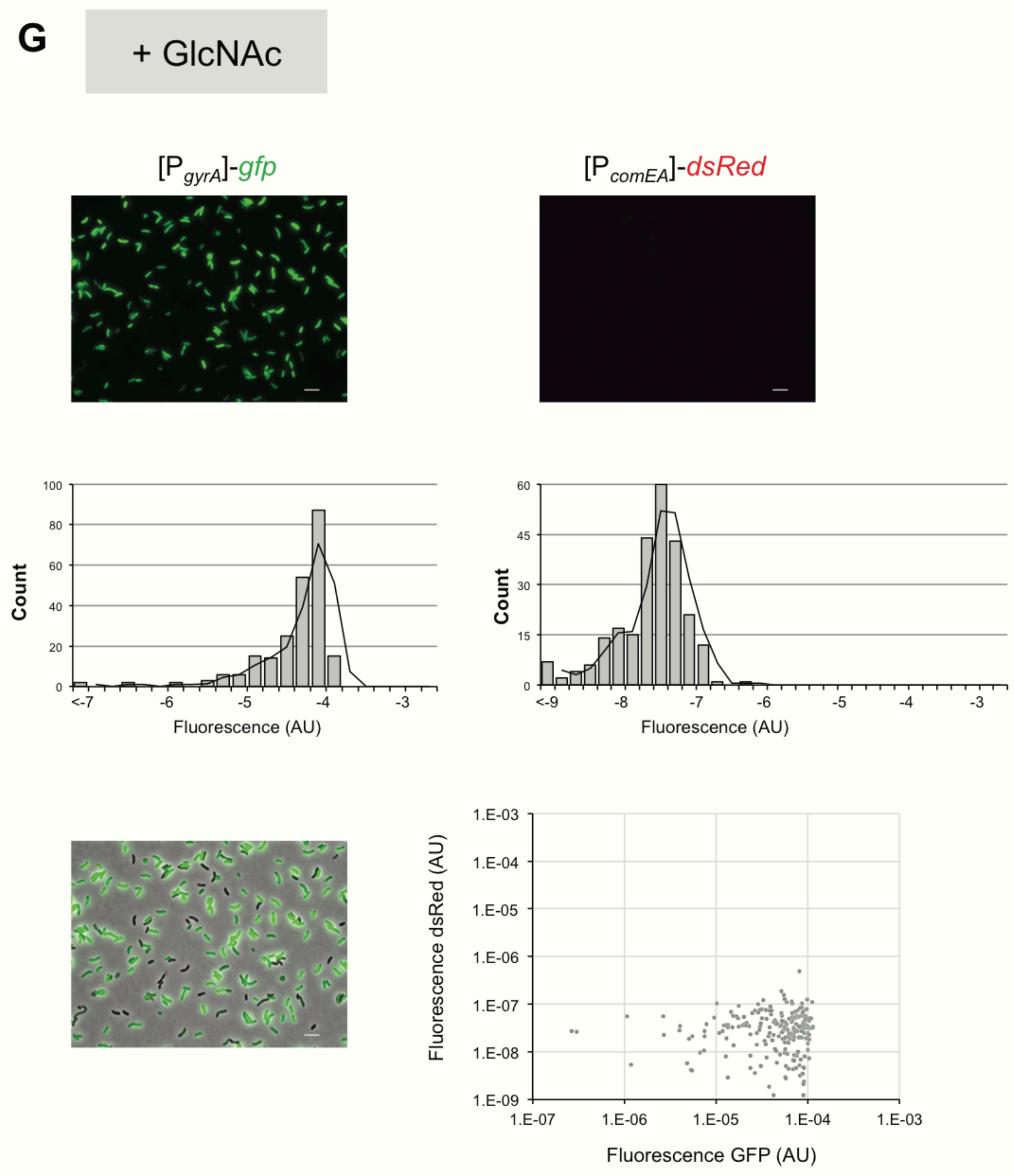

G
+ GlcNAc
[P gyrA]-gfp
[P comEA]-dsRed
Count
Fluorescence (AU)
Count
Fluorescence (AU)
Fluorescence dsRed (AU)
Fluorescence GFP (AU)

Fig. S2G

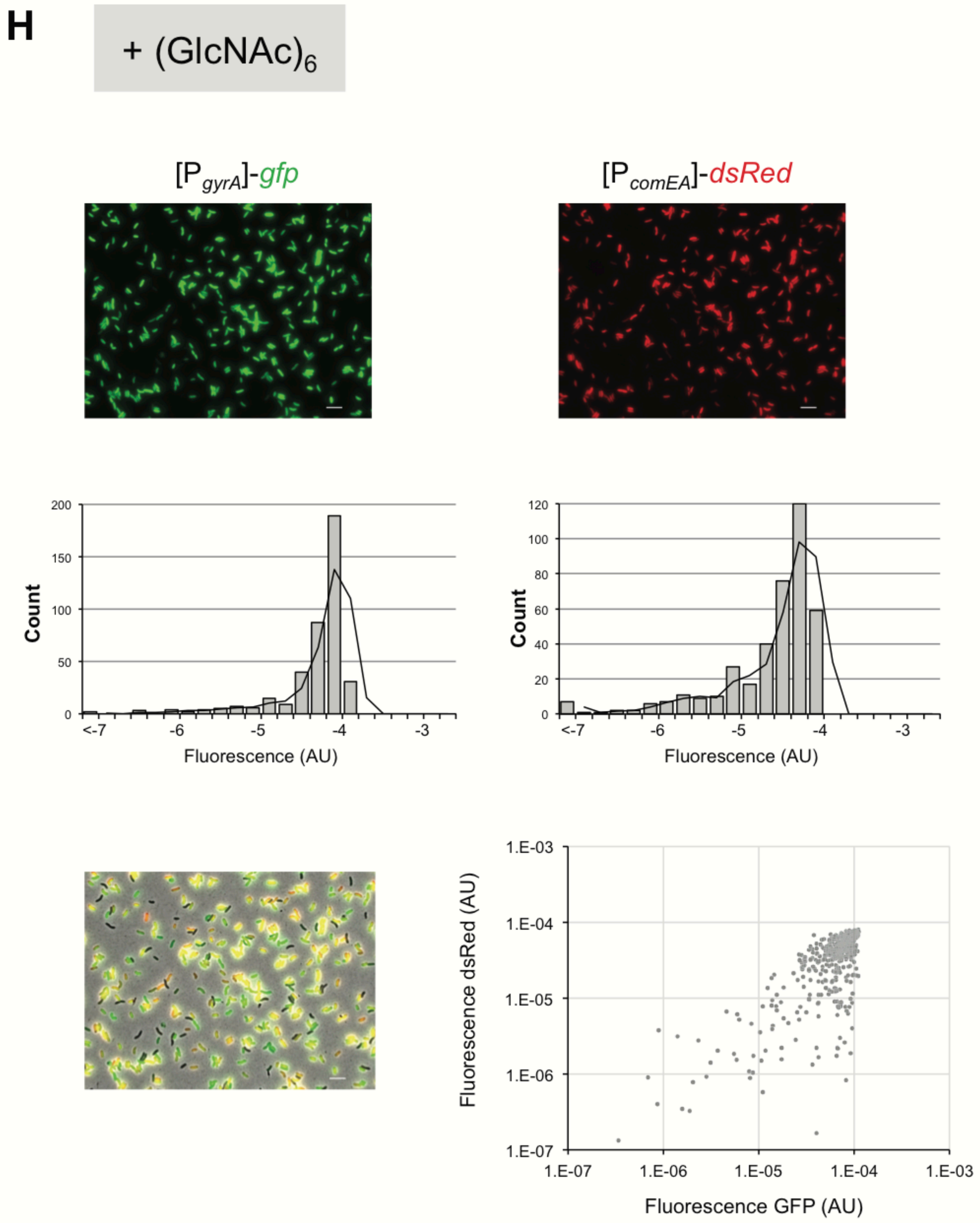
H
+ (GlcNAc)6
[PgyrA]-gfp
[PcomEA]-dsRed
200
150
100
50
0
Count
<-7
-6
-5
-4
-3
Fluorescence (AU)
120
100
80
60
40
20
0
Count
<-7
-6
-5
-4
-3
Fluorescence (AU)
1.E-03
1.E-04
1.E-05
1.E-06
1.E-07
Fluorescence dsRed (AU)
1.E-07
1.E-06
1.E-05
1.E-04
1.E-03
Fluorescence GFP (AU)

Fig. S2H
