# Supplementary material for: The Regulatory Network of Natural Competence and Transformation of Vibrio cholerae
Source: PLoS Genet. 2012 Jun 21;8(6):e1002778. doi: 10.1371/journal.pgen.1002778 (PMC3380833; doi:10.1371/journal.pgen.1002778)
Supplement: Figure S3 — Housekeeping genes are expressed in the majority of cells under homogeneous conditions. The indicated bacterial reporter strains were grown aerobically in DASW medium with GlcNAc6 as inducer of competence. Promoter-driven FP gene expression was either visualized by epifluorescence microscopy (images) or quantified for fluorescence intensities using flow cytometry (graphs below the fluorescence images). Scale bar in all images = 5 µm. (PDF) [file pgen.1002778.s003.pdf]

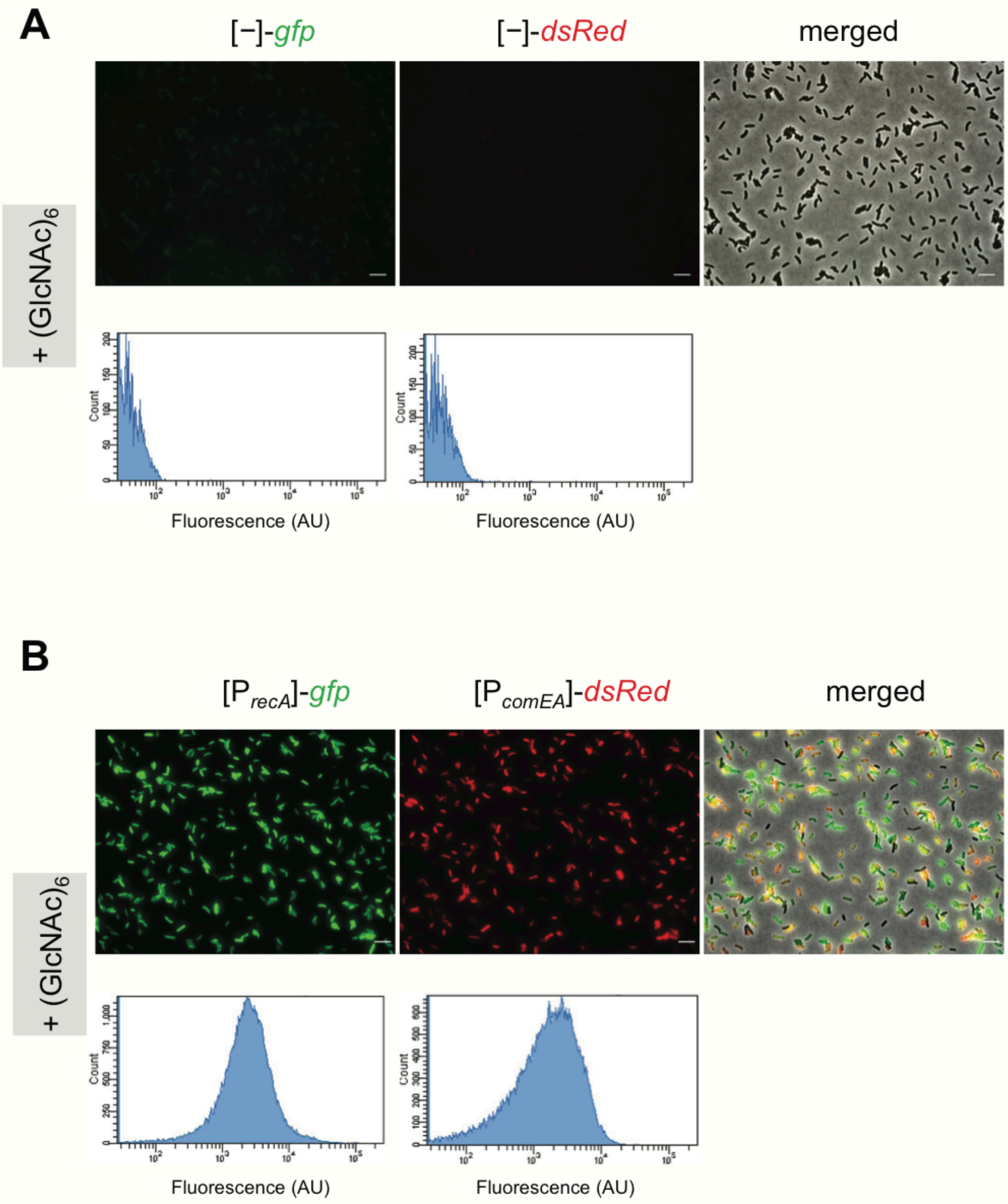
A
[−]-gfp
[−]-dsRed
merged
+ (GlcNAc)6
Count
Fluorescence (AU)
Fluorescence (AU)
B
[PrecA]-gfp
[PcomEA]-dsRed
merged
+ (GlcNAc)6
Count
Fluorescence (AU)
Fluorescence (AU)

Fig. S3A/B

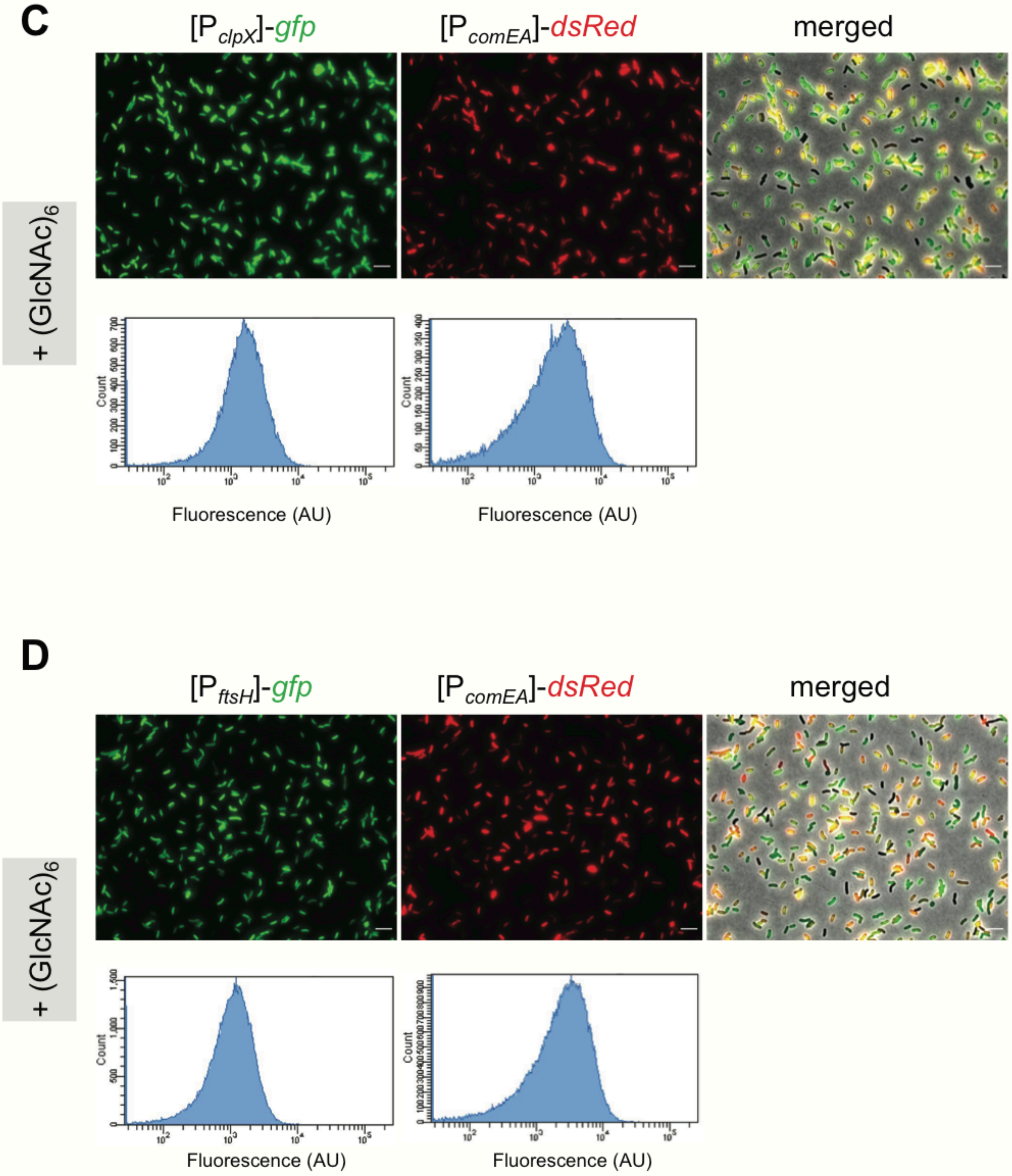
C
[PclpX]-gfp
[PcomEA]-dsRed
merged
+ (GlcNAc)6
Count
Fluorescence (AU)
Fluorescence (AU)
D
[PftsH]-gfp
[PcomEA]-dsRed
merged
+ (GlcNAc)6
Count
Fluorescence (AU)
Fluorescence (AU)

Fig. S3C/D
