# Supplementary material for: The Regulatory Network of Natural Competence and Transformation of Vibrio cholerae
Source: PLoS Genet. 2012 Jun 21;8(6):e1002778. doi: 10.1371/journal.pgen.1002778 (PMC3380833; doi:10.1371/journal.pgen.1002778)
Supplement: Table S1 — Primers used in this study. (DOCX) [file pgen.1002778.s009.docx]

**TABLE S1. Primers used in this study**

| **Primer name** | **Sequence**  (given in 5’ to 3’ direction) | **Comments** |
| --- | --- | --- |
| NEW-VC2433-SacI#1 | CGCGAGCTCAAATGACCTATCAACTGCAAGTGC | PCR of flanking regions of gene VC2433 to create knock-out plasmid  pGP704-28-SacB-ΔcpdA |
| NEW -VC2433#2 | CTTGGCGACTTACTCGGGTGTGTGGTCTTGCGAAATATCC |  |
| NEW -VC2433#3 | ACACCCGAGTAAGTCGCCAAGCACAGTAATGTGAAAGCC |  |
| NEW -VC2433-XbaI#4 | CGCTCTAGATAACCGCCTAATGAACTTCCGACC |  |
| F_GFP-EcoRI/SmaI | GCCGAATTCCCGGGCCGCCATGTCCCTATAATGCGAC | PCR of promoterless *gfp* gene |
| R_GFP-AatII | CGGGACGTCAACGCTGCCCGAGATGCGCCGCG |  |
| F_dsRed-EcoRV/StuI | GCCGATATCAGGCCTTCACACAGGAAACAGCTATGACC | PCR of promoterless *dsRed* gene |
| R_dsRed-BamHI | GCCGGATCCGGGGATCCTCTAGAGTCAAAAGG |  |
| F_KanR | GCCGGATCCATCATGAACAATAAAACTGTCTGCTTAC | PCR of *aph* gene |
| R_KanR | GCCGGATCCTGCCAGTGTTACAACCAATTAACC |  |
| F-[VC1917]-200-up | CTGGCGGGTGTGATCAGTGTGTTG | PCR of *comEA* promoter region |
| P-[VC1917] reverse | PGATAGACCCTCATTTTGGTTGTTG |  |
| F_pilA-AvrII-XbaI | GCGCCTAGGTCTAGAGCACAGCGAAACAGCTCAATCCCG | PCR of *pilA* promoter region |
| p(VC2423+99+STOP) | CTAAATCATCAATTCAATTAAGGTAAAACC |  |
| F-P[VC0047] | GCGAGGTGATCGAGTTCGTGCTGTACACAG | PCR of *VC0047-50* promoter region |
| R-[VC0047]-EcoRI | CCGTAGAATTCGTCCGGATGCTGTCATTTGGGTC |  |
| F-EcoRI-[hapA] | CCAAGAATTCGGCTTTCTTATCGAGTATTCCTGC | PCR of *hapA* promoter region |
| R-XmaI-[hapA] | CCAACCCGGGTTCTCAATCCTAGAGATGTTGAATG |  |
| F-EcoRI-gyrA | CCAAGAATTCTTTAAGACGCAACCAAGGTCACAACC | PCR of *gyrA* promoter region |
| R-XmaI-gyrA | CCAACCCGGGAGAGCCATTATCCCTCTATAGTTTGATCG |  |
| F-EcoRI-recA | CCAAGAATTCGCGAACAAGTGCGTCAGCAAGCC | PCR of *recA* promoter region |
| R-XmaI-recA | CCAACCCGGGTACTCTCTCCGGATAGTCACTCTCAGG |  |
| F-EcoRI-ftsH | CCAAGAATTCAATCCGTAAACCGGACTCCTCTCG | PCR of *ftsH* promoter region |
| R-XmaI-ftsH | CCAACCCGGGTCACTCAAGGTGTAAGCCTCGCGATAAC |  |
| F-EcoRI-clpX | CCAAGAATTCATACAGGCCAGCCAATCGAAGTGATTG | PCR of *clpX* promoter region |
| R-XmaI-clpX | CCAACCCGGGTCGCTAACCTCTTAGCCTTTACTCAC |  |
| VC1879-up-EcoRI | CCAAGAATTCAGCCATAATCATTTGGCTGGGCAG | PCR of *comEC* promoter region |
| VC1879-down-XmaI | CCAACCCGGGGAAAATTCATTAAACGGTTTATGCC |  |
| XmaI-comEA for gfp | GTTGCCCGGGGATAGACCCTCATTTTGGTTGTTG | PCR of [P*_comEA_*]-[P*_pilA_*] (“promoter-swapping”) |
| StuI-pilA for dsRed | GTTGAGGCCTCTAAATCATCAATTCAATTAAGG |  |
| araC-rev_XmaI | GCGCCCGGGATTCTGCAAACCCTATGCTACTCCG | PCR fragment araC-P*_BAD_*-*tfoX*-amplified from pBAD-*tfoX*-stop |
| pBADterm_bck_NotI | AACCCGCGGCCGCTCCCGGCGGATTTGTCCTACTCAGG |  |
| gyrA-157-fwd | AATGTGCTGGGCAACGACTG | qRT-PCR for *gyrA* transcription |
| gyrA_332_bwd | GAGCCAAAGTTACCTTGGCC |  |
| qRT_recA_fwd | CTCTGTCTCTGGATATCGCG | qRT-PCR for *recA* transcription |
| qRT_recA_bwd | GCATAAACCGGATCCAGTGC |  |
| qRT_ftsH_fwd | TGGTCAAGGCCAGATTCAGG | qRT-PCR for *ftsH* transcription |
| qRT_ftsH_bwd | TTGCTCTTCAGGAGGCGTAC |  |
| qRT_clpX_fwd | GTTCGCAAACTGATCGCAGG | qRT-PCR for *clpX* transcription |
| qRT_clpX_bwd | CGGCTAGCACTTTTTTGGCG |  |
| NEW_pilA_283_fwd | ATTGCCCCAACAGCATCAGG | qRT-PCR for *pilA* transcription |
| NEW_pilA_453_bwd | TGTTGCACCTGCAGTACAGC |  |
| comEA_50_fwd | CGACATTACCGTTACTGGCC | qRT-PCR for *comEA* transcription |
| comEA_224_bwd | CCGTTGGCTTCTCGATAATCG |  |
| comEC_1029_fwd | GGTCGCGATTGTTCTCTACC | qRT-PCR for *comEC* transcription |
| comEC_1186_bwd | CCAAATTGTACAGAACTGCCG |  |
| pilM_218_fwd | CGATAGCAATCCCGGATAGC | qRT-PCR for *pilM* transcription |
| pilM_415_bwd | ACACTTGGAAGGTCGTCGTG |  |
| VC0047-573-fwd | CGAGGTAGCACAGTATCAAC | qRT-PCR for *VC0047* transcription |
| VC0047-742-bwd | GCTCCTCAAGATCGACTTCTG |  |
| dprA_115_fwd | ACGCCTAAGCAATGGTTGGC | qRT-PCR for *dprA* transcription |
| dprA_309_bwd | AATAAACAGGACGGGAGGCG |  |
| dns_276_fwd | GCAATGCTGGCAACAAGGTG | qRT-PCR for *dns* transcription |
| dns_443_bwd | CCATAGGTGACGCCATCAAC |  |
